# Chromatin accessibility analysis reveals regulatory dynamics and therapeutic relevance of Vogt-Koyanagi-Harada disease

**DOI:** 10.1038/s42003-022-03430-9

**Published:** 2022-05-26

**Authors:** Wen Shi, Jinguo Ye, Zhuoxing Shi, Caineng Pan, Qikai Zhang, Yuheng Lin, Yuanting Luo, Wenru Su, Yingfeng Zheng, Yizhi Liu

**Affiliations:** 1 State Key Laboratory of Ophthalmology, Zhongshan Ophthalmic Center, Sun Yat-sen University, Guangdong Provincial Key Laboratory of Ophthalmology and Visual Science, Guangzhou, 510060 China; 2 Research Unit of Ocular Development and Regeneration, Chinese Academy of Medical Sciences, Beijing, 100085 China; 3 Guangzhou Regenerative Medicine and Health Guangdong Laboratory, Guangzhou, 510005 China

**Keywords:** Autoinflammatory syndrome, Uveal diseases

## Abstract

The barrier to curing Vogt–Koyanagi–Harada disease (VKH) is thought to reside in a lack of understanding in the roles and regulations of peripheral inflammatory immune cells. Here we perform a single-cell multi-omic study of 166,149 cells in peripheral blood mononuclear cells from patients with VKH, profile the chromatin accessibility and gene expression in the same blood samples, and uncover prominent cellular heterogeneity. Immune cells in VKH blood are highly activated and pro-inflammatory. Notably, we describe an enrichment of transcription targets for nuclear factor kappa B in conventional dendritic cells (cDCs) that governed inflammation. Integrative analysis of transcriptomic and chromatin maps shows that the RELA in cDCs is related to disease complications and poor prognosis. Ligand-receptor interaction pairs also identify cDC as an important predictor that regulated multiple immune subsets. Our results reveal epigenetic and transcriptional dynamics in auto-inflammation, especially the cDC subtype that might lead to therapeutic strategies in VKH.

## Abstract (web summary)

Single-cell RNA-sequencing and chromatin accessibility analysis reveal transcriptomic and epigenomic changes between healthy patients and those affected by the systemic autoimmune disorder Vogt-Koyanagi-Harada disease.

## Introduction

Vogt–Koyanagi–Harada disease (VKH) is a systemic autoimmune disorder characterized by bilateral granulomatous uveitis with meningeal, auditory, and dermal manifestations^1^. It is one of the major sight-threatening uveitis entities in Asia and South America^2–4^. Aggressive systemic corticosteroids in combination with immunosuppressive agents remain the mainstay of treatment^5,6^, but a large proportion of patients progress and have a poor prognosis, leading to visual impairment, reduced quality of life, and even blindness. In addition, the undesirable side effects (e.g., hyperglycemia, osteoporosis, and obesity) related to the prolonged use of corticosteroids and immunosuppressive agents highlight the need to develop new therapeutic strategies with fewer complications and less risk of treatment failures^7–9^.

A better understanding of how pathogenic networks in immune cells influence inflammation is a prerequisite for the treatment success of VKH. Previous studies have shown the involvement of T cells (especially, T helper 17 [Th17] and T helper 1 [Th1] cells) as a part of the systemic inflammatory process in animal experimental autoimmune uveitis (EAU) models and in blood samples of patients with VKH^10–12^. Th1 cells were the first T cell subsets considered to be the etiologic agent of VKH because of the cytotoxicity against melanocytes^13,14^. Several reports have implicated Th17 cells in the pathogenesis of VKH disease via IL-23/IL-17 pathway^15,16^. Recent single-cell RNA study has provided insight into the atlas of peripheral monocytes in VKH patients and how interferon-stimulated gene changes within monocytes reflects disease activity^17^. However, the role of other immune cell subtypes and their underlying epigenetic dysregulation in the pathogenesis of VKH has not been previously documented.

Single-cell assays for transposase-accessible chromatin sequencing (scATAC-seq) has emerged as a novel approach to delineate single-cell-specific epigenomic regulatory landscapes^18^. This technology enables genome-wide identification of cell-type-specific *cis*-elements, mapping of disease-associated enhancer activity, and inference of transcription factor (TF) binding and activity at a single-cell resolution^19^. In the current study, we aimed to delineate a multiomic landscape in peripheral blood mononuclear cells (PBMCs) derived from healthy individuals and patients with VKH based on an integrative analysis of single-cell RNA sequencing (scRNA-seq) and scATAC-seq datasets. We revealed a wide range of epigenomic and transcriptomic changes in healthy subjects and patients with VKH disease. Notably, we identified conventional dendritic cells (cDCs) as an important regulator of the pro-inflammatory state and revealed that RELA might be a key transcription factor in cDCs that is associated with highly inflammatory states and with poor prognosis. This study offers insights into therapeutic options for VKH and similar autoimmune diseases.

## Results

### Single-cell chromatin accessibility and transcription sequencing workflow

**Fig. 1:**
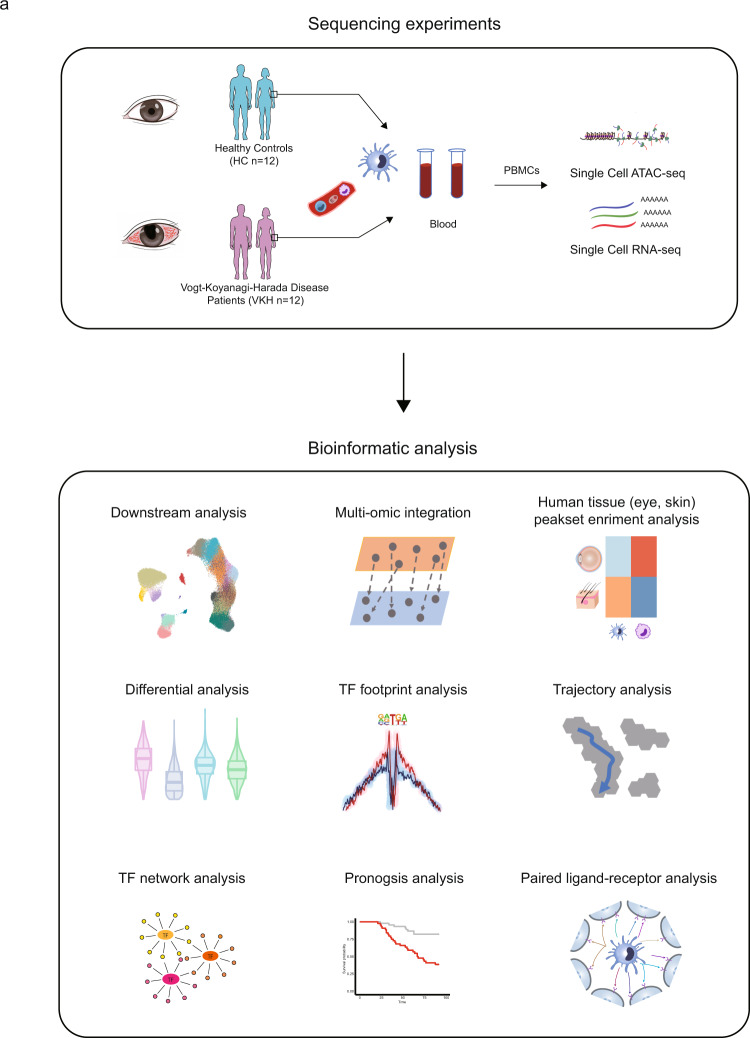
The single-cell multiomic experimental design. **a** Schematic representation of the single-cell profiling of PBMCs from healthy controls (*n* = 12) and VKH disease patients (*n* = 12) in this study, sequencing experiments and downstream bioinformatic analyses. All data are aligned and annotated to hg38 reference genome.

We isolated the nuclei and RNA from sex, age-matched individual PBMC samples from healthy individuals and VKH patients. In the first cohort, the PBMCs were obtained from patients diagnosed with acute VKH disease (*n* = 12) and the same healthy control (HC) group (*n* = 12) (Fig. 1a and Supplementary Table 1). Both nuclei and RNA were processed through the 10× Genomics platform using the standardized scATAC-seq and scRNA protocols, respectively. The scATAC-seq libraries were sequenced, the reads were de-multiplexed, and the fragments were aligned to the human reference genomes and de-duplicated using Cell Ranger ATAC. The scRNA-seq libraries were sequenced, demultiplexed and aligned to the human reference genomes and de-duplicated using Cell Ranger. The scATAC-seq data were analyzed using ArchR^20^, whereas the scRNA-seq datasets were processed using Seurat^21^. All these data were further analyzed after stringent quality control filtration, the thresholds of the scATAC-seq and scRNA-seq are described in the Methods (Supplementary Fig. 1a–c). For both scATAC-seq and scRNA-seq dataset, we conducted a harmony-based batch correction^22^ on each dataset. This allowed for meaningful downstream integrated analyses (Supplementary Fig. 2a-h and Methods). After quality-control filtering, we retained 133,140 cells for the scATAC-seq and 195,948 cells for the scRNA-seq analysis.

### Identification of cell types in healthy blood using scATAC-seq

**Fig. 2:**
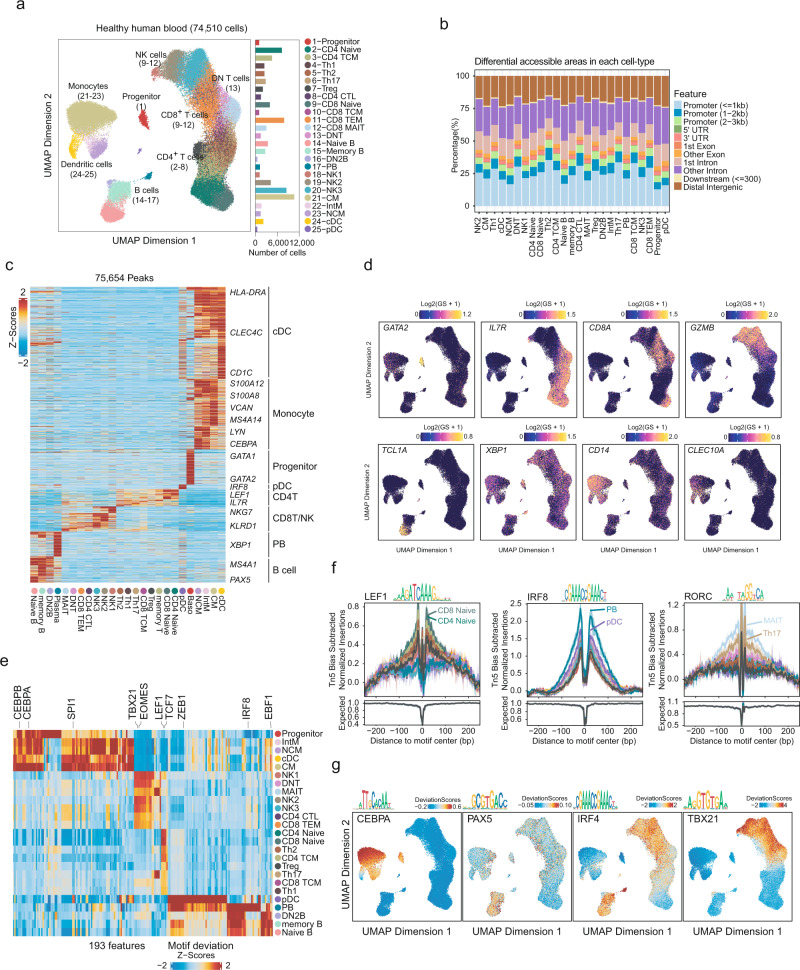
Single-cell chromatin landscape of health human peripheral immune cell subsets. **a** UMAP projection of 74,510 scATAC-seq profiles of peripheral blood immune cell types from 12 healthy controls. Dots represent individual cells, and colors indicate immune cell types (labeled on the right). Bar plot indicates the number of scATAC-seq profiles in each cell types. **b** Bar plot of annotated DAR locations for each cell type. **c** Heatmap of Z-scores of 75,654 cis-regulatory elements in scATAC-seq peripheral blood cell types derived from Fig. 2a. Gene labels indicate the nearest gene to each regulatory element. **d** UMAP projection colored by gene activity scores for the annotated lineage-defining genes in HC group of scATAC-seq dataset. The minimum and maximum gene activity scores are shown in each panel. **e** Heatmap representation of chromVAR bias-corrected deviations in the most variable TFs across all healthy immune cell types. **f** TF footprints with motifs in the indicated scATAC-seq healthy immune cell types. The Tn5 insertion bias track is shown below. **g** UMAP projection of scATAC-seq peripheral blood profiles colored by chromVAR TF motif bias-corrected deviations for the indicated factors. All data are aligned and annotated to hg38 reference genome.

To establish a baseline peripheral immune cell normal chromatin profile, we first identified 74,510 cells from healthy individuals, with an average of 9,047 uniquely accessible fragments per cell (Fig. 2a). We performed latent semantic indexing (LSI) for dimensionality reduction and harmony-based batch correction and applied Seurat to identify clusters^21^. Using these approaches, we identified 25 major scATAC-seq clusters, which were then visualized using uniform manifold approximation and projection (UMAP) (Fig. 2a). We first compared the differentially accessible chromatin regions (DARs) for each cell subset and applied ChIPseeker^23^ to annotate the distribution of the DARs in the genome. As expected, the distribution of the peak regions was relatively conserved across the different cell types, and the majority of the peaks were located in a promoter region within 3 kb of the nearest transcriptional start site (Fig. 2b).

To comprehensively describe the heterogeneity of the immune cell subsets in the PBMCs, we created a workflow to identify cell type and cell state signatures from scATAC-seq profiles, with reference to the gene expression/ATAC profiles for the cell types/subpopulations identified previously. We identified 75,654 cis-regulatory elements (CREs) across all the clusters and revealed cell type-specific cis-elements. By applying peak annotation analysis from ChIPseeker^23^ to identify the nearest genes to a peak, we could identify the cis-elements within a single gene locus. For example, Fig. 2c shows some of the known gene signatures for cDC (e.g., *HLA-DRA*^24^, *CLEC4C*^24^, and *CD1C*^24^), monocytes (e.g., *S100A12*^25^, *S100A8*^26^, *VCAN*^27^, *MS4A14*^28^, *LYN*^29^, and *CEBPA*^24^), progenitors (e.g, *GATA1*^19^ and *GATA2*^19^), plasmacytoid DCs (pDC) (*IRF8*^24^), CD4^+^ T cells (CD4T) (*LEF1*^30^ and *IL7R*^30^), CD8^+^ T cells (CD8T)/natural killer cells (NK) (*NKG7*^31^ and *KLRD1*^31^), plasma B cells (PB) (*XBP1*^32^), and B cells (*MS4A1*^33^ and *PAX5*^19^).

The sparsity of single-cell cis-element information prompted us to use gene activity scores (GAS) for cell type annotations in the scATAC-seq profiles. We utilized this analytical approach to confirm the cis-element-defined cluster identities and further classify the immune cell subpopulations^20^. In agreement with the surface phenotypes identified using the CRE approach, the progenitor cells showed a high GAS in the *GATA2*^19^ and naïve T cells in *IL7R*^30^ (Supplementary Fig. 3c, S4a, b). The high GAS on the surface markers CD8 and granzyme B (*GZMB*) further identified cytotoxic immune subsets^31^, including CD8T and NKs. The high GAS of *TCL1A* identified naïve B cells (Fig. 2d, Supplementary Fig. d, S4c, d)^33^. The GAS analysis across all clusters enabled the identification of phenotypically distinct cell subsets for dividing NK cells into three subsets. *NCAM1*^high^*FCGR3A*^low^*B3GAT1*^low^ NK cells were defined as early NKs (NK1), *NCAM1*^low^*FCGR3A*^high^*B3GAT1*^low^ NK cells were intermediate NKs (NK2), and *NCAM1*^low^*FCGR3A*^high^*B3GAT1*^high^ NK cells were late NKs (NK3)^34^ (Supplementary Fig. 3e, S4e, f). We were also able to identify myeloid subsets into monocytes (including classical monocytes [CM], intermediate monocytes [IntM], and non-classical monocytes [NCM]) and dendritic cells (including pDC and cDC) (Supplementary Fig. 3f, S4g, h). CM cells were *CD14*^+^*FCGR3A*^-^, IntM were *CD14*^+^*FCGR3A*^+^ while NCM were *CD14*^-^*FCGR3A*^+^^35^. 12 T cell subsets were also identified based on the GAS analysis: CD4^+^ naive T cells (CD4 Naive), CD4^+^ central memory T cells (CD4 TCM), T regulatory cells (Treg), T helper 2 cells, Th17 cells, cytotoxicity CD4^+^ T cells (CD4 CTL), CD8^+^ naive T cells (CD8 Naive), CD8^+^ central memory T cells (CD8 TCM), CD8^+^ effector memory T cells (CD8 TEM), CD8^+^ mucosal-associated invariant T (MAIT) cells and double-negative T cells (Supplementary Fig. 3c, S4a, b)^36,37^. In B cells, we identified *TCL1A*^+^ naive B cells (naive B), *CD19*^+^*ITGAX*^+^*TBX21*^+^*PDCD1*^+^*CXCR5*^low^*CR2*^-^double-negative 2 B cells (DN2B), memory B cells, and *XBP1*^+^ *CD38*^+^ PB^33,38,39^ (Supplementary Fig. 3d, S4c, d).

In addition to assessing the CREs and GAS for key lineage identification, we also measured chromatin accessibility at cis-elements sharing a TF binding motif using chromVAR^40^. In this approach, we incorporated both the TF footprints and TF deviation scores to further annotate and/or validate the rare cell subsets. For example, we identified the pDCs based on the enriched TF deviation scores of the IRF8 factor motif (Fig. 2e)^24^. The naïve T cells showed the activity of the T cell lineage-determining factor LEF1, consistent with the results from the TF footprint analysis (Fig. 2f)^19^. Surprisingly, we noticed that MAIT cells shared and even had a higher activity on the RAR-related orphan receptor (ROR) family than that of the Th17 cells, which are known to be key regulators in autoimmune diseases^37^ (Fig. 2f, Supplementary Fig. 3g). As expected, the TF deviation scores for PAX5, a lineage-determining factor for B cells^41^, were increased in all the B cell subsets. It is also important to note that DN2B showed the unique activity of TBX21, whereas IRF4 was active in the remaining B cell subsets (Fig. 2g). DN2B cells have been previously documented as an exhausted memory B cell subset^39^. In myeloid cells, we found that cDC showed higher activity for SPI1^24^, whereas CM showed higher activity for CEBPA^24^ (Supplementary Fig. 4i). Collectively, our approach allows for the analysis of chromatin accessibility in both common and rare cell types from human peripheral blood.

### Multi-omics analysis of the peripheral immune-cell profiling

**Fig. 3:**
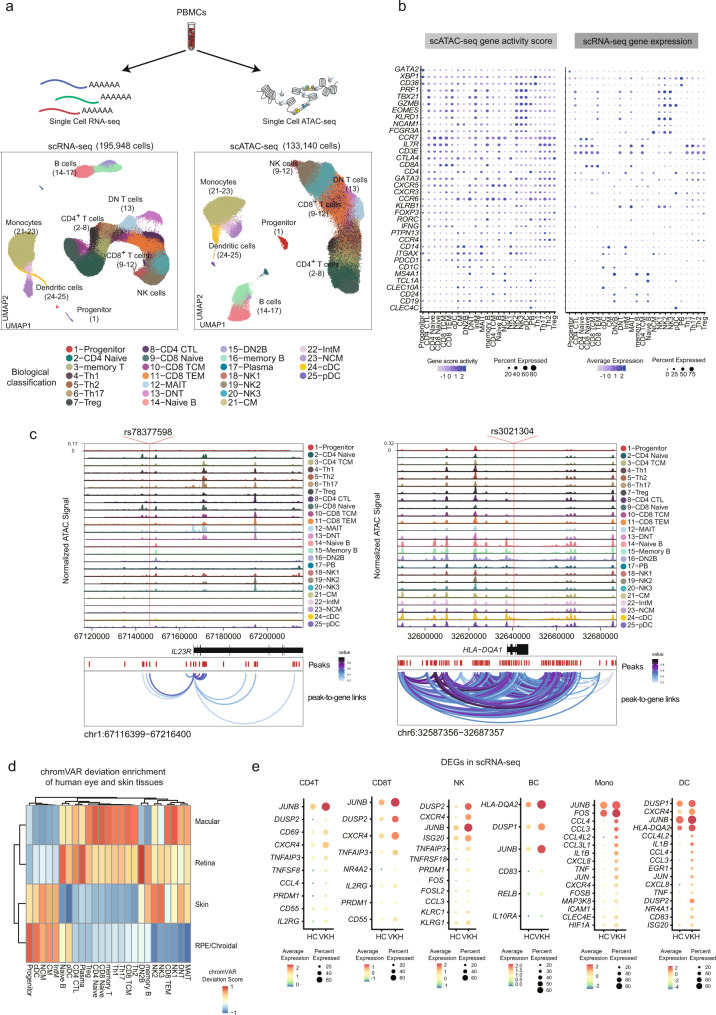
Overview of the immune-cell epigenetic and transcriptional landscape of PBMCs from VKH patients and healthy human. **a** Schematic for Multi-omics integration strategy for processing the scATAC-seq dataset and scRNA-seq dataset. **b** Dot plots of gene activity scores (left) and gene expression (right) of the marker genes in scATAC-seq and scRNA-seq dataset. The dot size indicates the percentage of the cells in each cluster in which the gene of interest. The standardized gene activity score level (left) and gene expression level (right) were indicated by color intensity. **c** Cis-regulatory architecture at the following GWAS loci and cell types in PBMCs: *IL23R* and *HLA-DQA1*. Only connections originating in the loci with peak-to-gene accessibility above 0.2 are shown. **d** ChromVAR deviation enrichment of the peakset of human tissues (including eyes and skins) from ATAC-seq and CHIP-seq dataset from HCs against the scATAC-seq dataset from healthy peripheral blood cell populations. **e** Dot plots of the expression level of the differential genes between normal and VKH CD4^+^T cells, CD8^+^T cells, nature killer cells, B cells, monocytes and dendritic cells in scRNA-seq dataset. All data are aligned and annotated to hg38 reference genome.

To study the PBMCs of VKH patients, we first integrated the VKH and the HC dataset and performed unbiased iterative clustering followed by Harmony-based batch correction on each sample in HC groups and VKH groups (Supplementary Fig. 2a-d). We then used the above-mentioned cell type identification pipeline to identify 25 immune subsets in 195,948 cells (Fig. 3a). Next, we processed the scRNA-seq data of VKH and HC groups and corrected the batch effect for each sample using Harmony-based batch correction (Fig. 3a). We manually annotated the 25 cell types in scRNA-seq dataset based on gene expression of the marker genes, which were consistent with the ones for our scATAC-seq data, to minimize the differences in cell-type composition between the two sequencing methods (Fig. 3a, Supplementary Fig. 5a–c). Next, we sought to illustrate the epigenetic regulation in the VKH patients. We utilized the recently developed method^21^ that identifies pairwise correspondences (called “anchors”) between single cells across two different types of datasets and projects their transformation into a shared space (Supplementary Fig. 6a–e)^42^. The whole procedure was parallelized and separately aligned using ArchR^20^ and Seurat^21^ by dividing each cell into smaller groups (see Method). This approach allowed us to integrate the gene expression data from the scRNA-seq data to the scATAC-seq data by mapping the gene score and gene expression to generate an integration matrix with gene expression in the scATAC-seq dataset (Supplementary Fig. 6a–e). As expected, the GAS and gene expression were highly consistent and could distinguish the cell types identified (Fig. 3b). The frequencies of the immune cell subsets between the HC and VKH groups were comparable (Supplementary Fig. 6f).

Our datasets also allowed us to dissect the mechanisms behind causal risk variants previously identified from genome-wide association studies (GWAS) and to identify disease-relevant cell types related to these loci. It was previously proposed that some of the disease-causing loci, while residing in noncoding regions^43^, could exert their effects by altering gene expression via perturbation of the TF binding sites and regulatory element function^44^. We collected known VKH GWAS loci reported in previous publications and mapped the disease-related single-nucleotide polymorphisms (SNPs) in the cis-elements for each cell^45^. Two variants, rs78377598 and rs3032304, were located within the *IL23R* and *HLA-DQA1* loci, respectively (Fig. 3c). The *IL23R* locus is highly accessible in MAIT cells, while *HLA-DQA1* is highly accessible in cDCs. These results may be informative for inferring the cellular impact of disease variants in these loci.

We also explored the potential mechanisms explaining how immune dysregulation results in organ damage in human ocular tissues and human skin. We used previously published ATAC-seq and CHIP-seq datasets of human eye tissues, including retina, macula, and retinal pigment epithelium (RPE)/choroid^46^, and human skin^47^ for cell-type-specific peak enrichment in our scATAC dataset (Fig. 3d, Supplementary Fig. 6g). We found that cDCs and monocytes subsets were mainly enriched in the RPE/choroid and skin. This might have implications in the pathogenesis of VKH, as the autoimmune attack is known to affect pigmented tissues, which results in vitiligo and ocular depigmentation^48^. In addition, we also revealed an enrichment of the CD4^+^ T cells in the retina and macular region as well as MAIT cells in the skin, retina, and macular regions (Fig. 3d).

Next, we analyzed the differential gene expression (DEG) on scRNA-seq dataset between the six main cell types by comparing the HC with VKH (Fig. 3e). We noticed that the T cells were activated in the VKH patients, with *CD69*^49^, *JUNB*^50^, and *CXCR4*^51^ being highly expressed (Fig. 3e). *TNFAIP3* was both highly upregulated in the T cells, which has been reported to be a common predisposing gene for autoimmune diseases, including VKH^52^. The NKs in VKH showed a higher cytotoxic capacity and higher chemokine levels, with upregulation of *ISG20*^49^, *DUPS2*^49^, *CCL4*^53^, and *CCL3*^53^, as compared to that in the HC (Fig. 3e). Moreover, the B cells also had an enhanced antigen-present function with an increased expression of *HLA-DQA2*^54^and *CD83* ^55^ with upregulation of genes of the nuclear factor kappa B (NF-κB) family and of the activator protein (AP-1) family (Fig. 3e)^55^. Myeloid cells in VKH were also identified as the main pro-inflammatory factor in patients with VKH disease. In the monocyte population, we found that the genes (e.g., *IL1B*^17^, *TNF*^17^, *CCL3*^17^, *CCL4*^17^, and *ICAM1*^56^) related to cytokines, chemokines, and adhesion were upregulated (Fig. 3e)^17^. Notably, *HIF1A* encodes the hypoxia-inducible factor (HIF) protein, which is also highly expressed in monocytes in VKH. Finally, for the DC subset, which is known as the key antigen presenter in immunity, they were more mature, with a higher capacity for antigen presentation by *CD83*^55^, as compared to the other subsets, and *HLA-DQA2* was upregulated in the patients (Fig. 3e). In addition, DCs acted as pro-inflammatory players, with high expression levels of cytokine and chemokine genes (e.g., *TNF*^57^, *CXCL8*^57^, *JUN*^56^, *JUNB*^56^, *CCL3*^57^, *CCL4*^57^, *IL1B*^57^, and *DUSP2*^56^). In summary, our results demonstrate that immune cells in VKH patients are generally activated and proinflammatory.

### T cell subsets and response in VKH

**Fig. 4:**
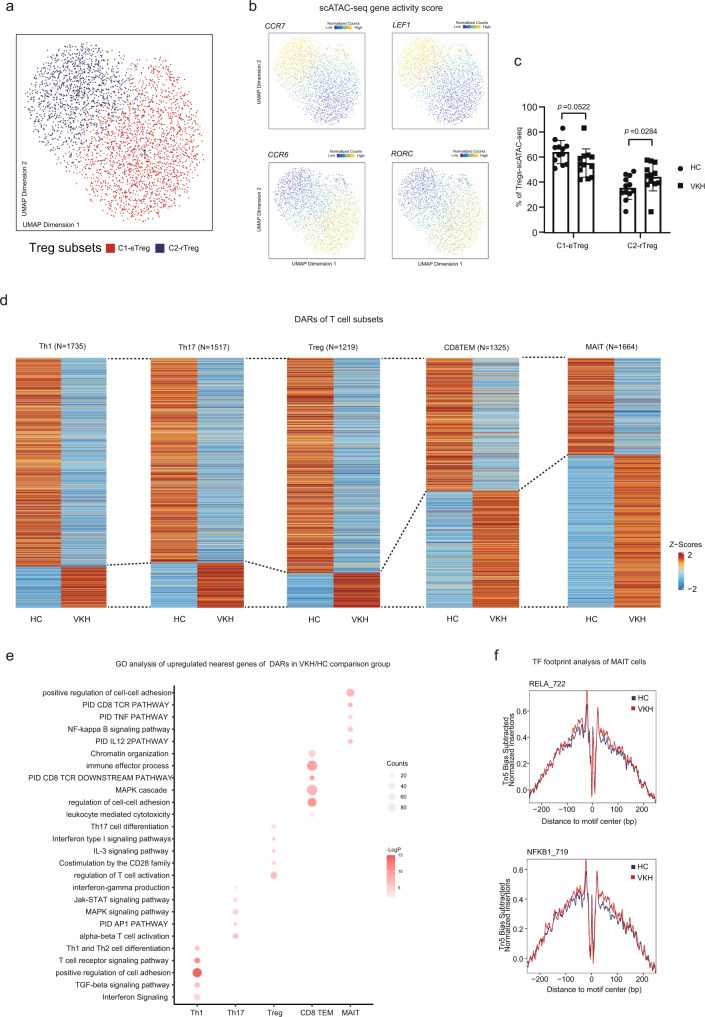
Epigenomic and transcriptional signatures of T cell subsets in VKH patients. **a** Subclustering UMAP of 3,182 CD4^+^ Treg. Dots represent individual cells, and colors indicate immune cell types (labeled on the below). **b** UMAP projection of CD4^+^ Treg colored by gene activity scores to the indicated gene. **c** Differences in the proportions of rTreg and eTreg among HC (*n* = 12) and VKH groups (*n* = 12). The adjusted *p* values were calculated using two-sided pairwise Wilcoxon test. **d** Heatmap of Z-scores of DARs in Th1, Th17, Treg, CD8TEM, and MAIT from HC and VKH. **e** Representative GO terms and KEGG pathways enriched in the nearest genes of upregulated DARs of Th1, Th17, Treg, CD8 TEM, and MAIT cells in the VKH/HC comparison group. **f** Comparison of aggregate TF footprints for RELA and NFKB1 in MAIT cells from HC and VKH. All data are aligned and annotated to hg38 reference genome.

To dissect the role of T cell subsets in VKH, we first compared the differences between T cell subsets among the HC and VKH patients in scATAC-seq. We first re-clustered the T regulatory (Treg) cells. This led to the identification of two subsets of Tregs with imbalanced frequencies between the VKH patients and HCs (Fig. 4a–c, Supplementary Fig. 5a). In cluster 1 (effector Treg [eTreg]), the effector genes such as *RORC*, *CCR8*, and *CCR6* were highly expressed. In cluster 2 (resting Treg [rTreg]), the remaining and naïve phenotype gene, such as *LEF1*, *TCF7*, and *CCR7* were expressed (Fig. 4b, Supplementary Fig. 5b)^58,59^. Although these gene differences were not observed between VKH and HC cells among the full Treg population in RNA expression, the rTreg demonstrated a significantly greater frequency, while eTreg showed a reduced frequency (of borderline statistical significance) in VKH in our scATAC-seq dataset^60^ (Fig. 4c).

We further compared the DARs in the five main T cell subsets (Th1, Th17, Treg, CD8^+^T effector memory (TEM), and MAIT) between the HC and VKH patients. The MAIT cells exhibited the largest number of peak changes among the T cell subtypes (Fig. 4d). In the VKH patients, *CD69*^37^, *JUNB*^61^, *CXCR4*^61^, and *PDRM1*^62^ were upregulated in the MAIT cells in our scRNA-seq dataset, suggesting the involvement of MAIT cell activation (Supplementary Fig. 7c). A Gene Ontology (GO) analysis of the nearest peak annotation of VKH-upregulated DARs showed that Th1 cells were involved in the interferon (IFN) and transforming growth factor (TGF)-beta signaling and T cell receptor signaling pathways, with higher accessibility to the *IFNG* locus (Fig. 4e, Supplementary Fig. 7d). In the Th17 cells, the AP-1 pathway, Janus kinase (JAK)-signal transducer and activator of transcription (STAT) signaling, and IFN-gamma production were activated, while Treg cells in the VKH were involved in IFN type I signaling pathway, CD28 family costimulation, interleukin (IL) 3 signaling, Th17 differentiation, and T cell activation, with higher accessibility to the *IL10* locus (Fig. 4e, Supplementary Fig. 7e). Among the CD8 T cells, the GO analysis of the CD8 TEM illustrated the regulation of cell–cell adhesion, enhanced CD8 T cell receptor (TCR) pathway and immune effector process, and enhanced cytotoxicity and MAPK cascade in VKH (Fig. 4e). The MAIT subset was involved in the MAPK signaling and CD8 TCR pathway and was positively related to cellular adhesion. Thus, MAIT cells may play an important role in adhesion molecules and integrins and in the migration of inflamed tissues (Fig. 4e). As expected, the MAIT cells were also associated with the activation of the cytokine pathway of the IL12 pathway, NF-κB signaling, and TNF pathway, consistent with their activated phenotype status. We further employed TF footprint analysis on the T cells to reveal the distinct TF footprints on the genomic DNA directly from VKH versus that in the HCs. Notably, the runt-related TF family members RUNX1 and TBX21 (also known as T-bet) were enriched in the Th17 cells, which are known to be involved in the production of pathogenic IFN-gamma production^63^ (Supplementary Fig. 7f). In the VKH patients, there was a more pronounced DNA occupancy of RELA and NFKB1 in the MAIT cells (Fig. 4f). The DNA occupancy was also identified higher activity of eomesodermin (EOMES) and TBX21 in the CD8 TEM cells as compared to that in the HCs (Supplementary Fig. 7g), indicating the enhanced effector states of CD8^+^ T cells. Overall, the T cells in the VKH patients exhibited activation phenotypes, with compositional and epigenomic alterations.

### CD14^+^ monocyte subsets and response in VKH blood

**Fig. 5:**
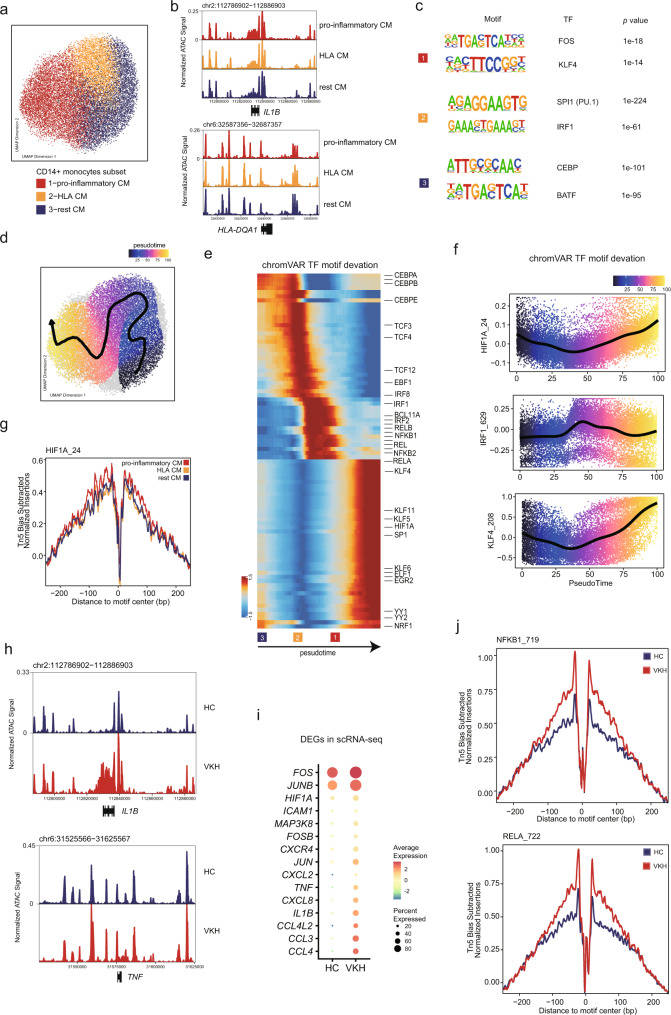
Epigenomic and transcriptional signatures of CD14^+^ monocytes subsets in VKH patients. **a** Subclustering UMAP of 20,054 CM cells. Dots represent individual cells, and colors indicate immune cell types (labeled on the below). **b** Genome browser tracks showing single-cell chromatin accessibility in the *IL1B* and *HLA-DQA1* locus. **c** TF motif enrichment analysis of cluster-specific sequences. **d** UMAP showing the lineage trajectory of CM ordered based on pro-inflammatory, HLA^+^ and rest states. Pseudotime values were overlaid on the UMAP embedding; the smoothed line and arrow represent the visualization of the trajectory path from the spline fit. **e** Heatmaps of the ordered TF motif accessibility across pseudotime in the CM (see Fig. 5d). The TF motif accessibilities are indicated by chromVAR TF-motif bias-corrected deviation. **f** chromVAR bias-corrected deviation scores for the indicated TFs across CM pseudotime. Each dot represents the deviation score in an individual pseudotime-ordered scATAC-seq profile. The line represents the smoothed fit across pseudotime and chromVAR deviation scores. **g** Comparison of aggregate TF footprints for HIF1A in CM subsets. **h** Genome browser tracks showing single-cell chromatin accessibility in the *IL1B* and *TNF* locus. **i** Dot plots of the expression level of the differential genes between normal and VKH in CM in RNA-seq dataset. **j** Comparison of aggregate TF footprints for NFKB1 and RELA in CM from HC and VKH. All data are aligned and annotated to hg38 reference genome.

The CD14^+^ monocytes have previously been recognized as pro-inflammatory players in VKH^17^. To investigate the enhanced inflammation epigenetic reprogramming in CD14^+^ monocytes, we reclustered the CM and observed three sub-clusters in the CM (Fig. 5a). Based on the peak accessibility, GAS and gene expression of *IL1B* and *HLA-DQA1*^17^, the proinflammatory CM characterized by the highest expression on *IL1B* and the HLA CM showed high expression on human leukocyte antigen (HLA)-related gene, and the remaining CM showed high expression on the *S100A8* and *VCAN* loci (Fig. 5b, Supplementary Fig. 8a-c)^64^. We further compared the DARs between each cell state and noticed that the marker peaks of each state were different (Supplementary Fig. 8d). We used the differential ATAC-seq peaks as an input to conduct TF motif enrichment analysis^65^ and identify the TFs associated with their differentiation programs (Fig. 5c). We noticed that pro-inflammatory CM relied on the AP-1 family and Krüppel-like family (KLF) motifs, which are essential for monocyte activation and maturation^66,67^. As for the HLA CMs, we identified increased accessibility of the IRF1 and ETS family TFs, SPI1 (also known as PU.1), which are related to IFN stimulation and major histocompatibility complex (MHC) class II gene expression (Fig. 5c)^68–70^. The rest of the CM was enriched in CCAAT/enhancer-binding protein (CEBP) family members and basic leucine zipper ATF-like TF (BATF) (Fig. 5c).

To understand the developmental dynamics of pro-inflammatory monocytes, we constructed a cellular lineage trajectory of CD14^+^ monocytes based on their differentiation states, which progressed from rest to a pro-inflammatory state. We generated ordered single cells (termed as ‘pesudotime’) based on our multi-omic dataset (Fig. 5d, Supplementary Fig. 9a-b). The dynamic TF motif activities across the trajectories were consistent with their differentiation states (Fig. 5e). For instance, IRF1 activity was observed in the HLA CM, consistent with the result of our motif enrichment analysis, followed by the sequential activity of CEBPA, SPI1, and FOS, recapitulating the known order of their functions in rest CM, HLA CM, and pro-inflammatory CM, respectively (Fig. 5e, f). In addition, HIF1A and KLF TFs are also activated in the pro-inflammatory CM^71^. Moreover, the TF footprint analysis showed changes surrounding the HIF1A binding sites in the pro-inflammatory CM but not in the other CM subsets, suggesting the possibility of the role of HIF1A as a key transcription factor driving monocyte maturation and inflammation (Fig. 5f, g). Together, our results revealed epigenetic reprogramming in the development of monocytes.

Next, we analyzed the differential peaks and genes of CM between the HC and VKH patients. Although we did not observe changes in the frequency of CMs, the difference in chromatin accessibility and gene expression was notable between HC and VKH, which was consistently shown in each dataset (Fig. 5h, i, Supplementary Fig. 4b, S9c). In scRNA-seq dataset, we analyzed the upregulated DEGs and showed that CMs in VKH were characterized by various cytokine and chemokine genes (*CCL3L1*, *CCL4*, *CCL3*, *IL1B*, *TNF*, *CXCL8*, and *CXCR4*)^17^, with enhanced cellular adhesion capacity (*ICAM1*)^56^. The high expression of *HIF1A* in CM suggesting the importance of HIF control in the inflammatory activity of monocytes. The TF footprints also showed an increase in chromatin accessibility in the footprint depth of the NF-κB family (e.g., RELA and NFKB1) in VKH, representing the highly inflammatory state of CMs (Fig. 5j). Collectively, our results shed light on the interdependence of innate immunity inflammation and hypoxic responses in VKH patients, showing that CD14^+^ monocytes might maintain a rapid inflammatory response through HIF1A-driven chromatin reprogramming immunity.

### Disease-specific TF regulatory patterns in the cDCs

**Fig. 6:**
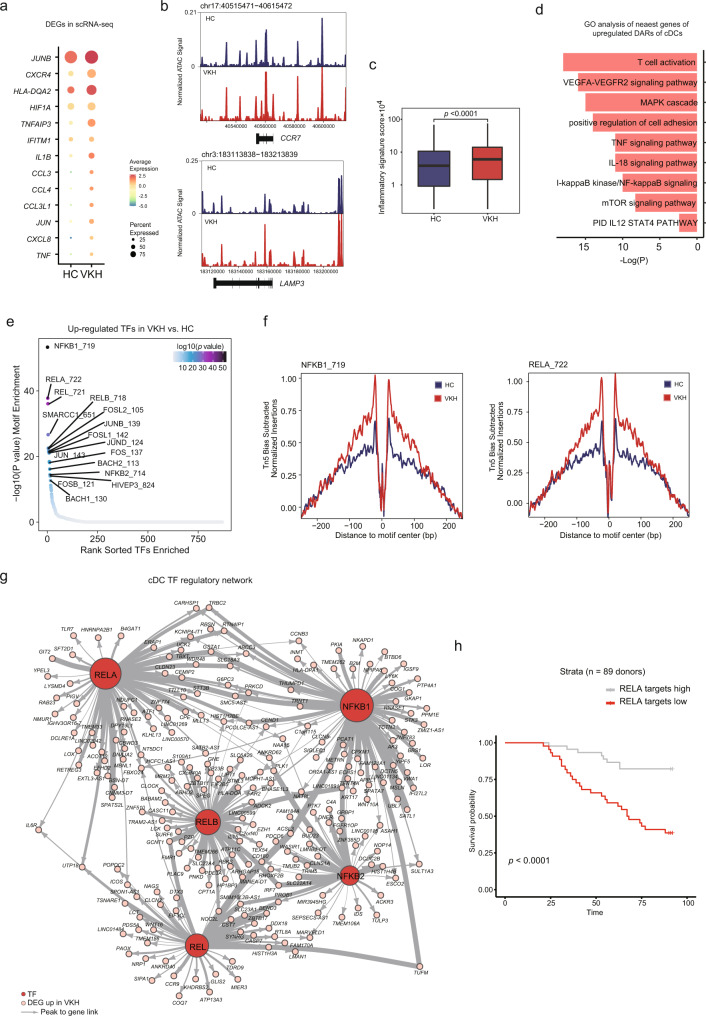
Epigenomic and transcriptional signatures of cDC subsets in VKH patients. **a** Dot plots of the expression level of the differential genes between normal and VKH in cDCs in scRNA-seq dataset. **b** Genome browser tracks showing single-cell chromatin accessibility in the *CCR7* and *LAMP3* locus. **c** Box plot of inflammatory signature score in all cells of each group. All *p* values were calculated using Kruskal-Wallis test. **d** Enrichment of biological processes associated with nearest genes of DARs in VKH compared to HC regions. **e** Visualization of TF binding motif enrichment analysis results for DARs in VKH compared to HC regions by using CIS-BP database from chromVAR. **f** Comparison of aggregate TF footprints for NFKB1 and RELA in cDC cells from HC and VKH. **g** TF regulatory network showing the NF-κB family and its potential target genes in VKH. The width of an edge indicates the peak to gene linkage correlation. **h** Kaplan–Meier curve for patients with VKH (*n* = 89) stratified by putative RELA-target genes (*n* = 328); average z score log2(expression) (log-rank test *p* < 0.001). All data are aligned and annotated to hg38 reference genome.

To further describe the potential function of cDCs in VKH, we conducted a differential analysis at the epigenomic and transcriptional levels (Fig. 6a, Supplementary Fig. 9d). In accordance with our scATAC-seq data, the cytokine and chemokine genes (*IL1B*, *CCL3L1*, *CXCR4*) were upregulated and were more accessible in VKH than in the HCs, representing the activated states of DCs (Fig. 6a). In addition, we observed an increased expression of the HLA genes (*HLA-DQA2*) and increased accessibility of *LAMP3* and *CCR7*, indicating a mature and enhanced antigen-presenting capacity of cDCs (Fig. 6a, b).

To assess the pro-inflammatory state of cDCs in VKH, we utilized the marker genes of inflammatory *CD1C*^+^ DCs documented in a published study^72^ (Supplementary Table 2) to estimate the inflammation scores across all cDCs. By comparing VKH with HC, we identified higher inflammation scores in VKH, which exhibited a strong potential to secrete immune mediators and lead to autoimmune disease (Fig. 6c).

To further elucidate the pathogenic pathways and regulators involved in VKH, we next utilized the nearest DAR genes for our GO and motif enrichment analysis (Fig. 6d, e). The top signaling pathways of cDCs in VKH included T cell activation and the IL12-STAT4 pathway, indicating the capacity to activate adaptive immunity (Fig. 6d). We also identified pathways involved in DC activation, maturation pathways (involving MAPK cascade, NF-κB signaling, and TNF signaling), mammalian target of rapamycin (mTOR) signaling pathway, and pathways for cell adhesion (Fig. 6d). Interestingly, we also revealed a pro-angiogenic VEGFA-VEGFAR2 signaling pathway and IL18 signaling that are implicated in the angiogenesis process were also enriched in cDCs (Fig. 6d). By employing the motif analysis of the DARs, we identified a significantly enriched NF-κB family (NFKB1, NFKB2, RELA, REL, and RELB) in VKH (Fig. 6e). The chromatin accessibility of the AP-1 family motifs (JUNB, FOSL1, JUND, and FOS) and BTB and CNC homology (BACH) family motifs (BACH1 and BACH2) were also upregulated (Fig. 6e). In accordance with the motif-enriched data, the cDCs in VKH showed notably higher occupancy in RELA and NFKB1 in our footprint analysis (Fig. 6f).

To illustrate the NF-κB-family-centered regulatory program network in the cDCs in VKH, we employed a recently established method to identify putative TF target genes based on the scATAC-seq and scRNA-seq data^73^ (see Methods, Supplementary Fig. 6d). First, we identified differentially linked peaks and genes. Next, the NF-κB family motifs were selected and assembled to identify the linked differential accessibility regions (Supplementary Fig. 10a). Finally, all the linked genes were combined to create a linkage score and the genes needed to exhibit differential expression and accessibility in the groups. Using this approach, we found 1372 genes regulated by the NF-κB family, containing distal elements in VKH (Supplementary Data 1). We further constructed an NF-κB family regulatory network based on the TFs and TF-targeted genes (Fig. 6g). For instance, one of the NFKB1 targeted genes in VKH, *SIGLEC1*, was previously reported to have genetic associations with autoimmune disease^74^. In summary, this approach provides a comprehensive regulatory network to unveil the role of the NF-κB family in cDCs.

To validate the role of activation of RELA in cDCs among the VKH patients, we included the RNA-seq data from Cohort 2, in which 89 VKH patients were recruited and had their peripheral blood drawn at baseline and at the three-month follow-up (Supplementary Data 2). We used the identified RELA target genes in VKH to stratify patients with different prognoses. We observed significantly decreased survival (*p* < 0.0001) in patients with a high RELA-target-gene signature (Fig. 6h). The NFKB1 target genes also showed a potential for stratifying VKH patients with different prognoses (*p* < 0.00012) (Supplementary Fig. 10b). Altogether, RELA is an important TF and acts as a prognostic predictor in VKH.

### cDC-centric cellular communication network

**Fig. 7:**
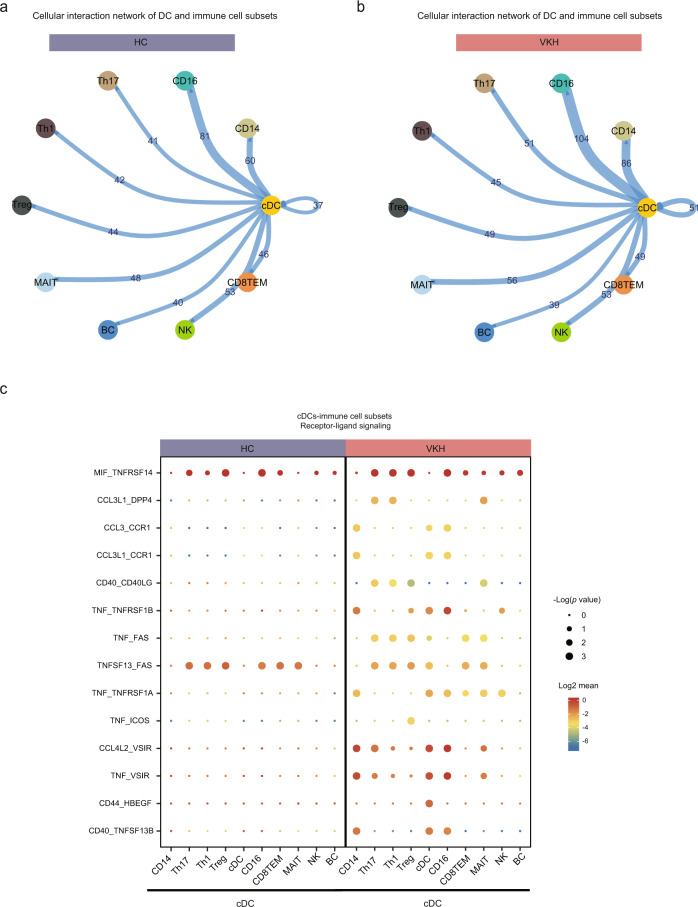
The cDC-centric cellular communications of peripheral immune cells in HC and VKH. **a** Network plot showing the changes in ligand-receptor interaction events between cDCs and indicated immune cell types in the HC group. Cell-cell communication and the number of ligands and receptors are indicated by the connected line. **b** Network plot showing the changes in ligand-receptor interaction events between cDCs and indicated immune cell types in the VKH group. Cell-cell communication and the number of ligands and receptors are indicated by the connected line. **c** Dot plot of predicted interactions between cDCs and indicated immune cell types in HC and VKH. Circle sizes indicated *p* values. The expression levels of the interacted genes were indicated by colors, scales on the right. All data are aligned and annotated to hg38 reference genome.

To identify the reciprocal communication between cDCs and other immune effector cell subsets, we surveyed the accumulated ligand/receptor interaction database CellPhoneDB^75^. VKHs had a stronger cell-cell interaction in cDCs than in HCs (Fig. 7a, b). Myeloid lineage clusters showed the highest capacity for cell–cell interactions (Fig. 7a, b). The cDCs shared the highest number of predicted interactions with monocyte subsets and even increased in patients with VKH disease. In line with the essential roles of cDCs and T cells as immune regulators in VKH, cDCs harbored ligand numbers 51, 45, 49, 56, and 49, with Th17, Th1, Treg, MAIT, and CD8TEM, respectively (Fig. 7b).

After examining the differentially expressed receptor–ligand pairs, we further identified enhanced immunomodulation in the cDCs in VKH (Fig. 7c). In terms of immunomodulation, we identified an increased interaction of cDCs with CD4T and CD8T through the prediction of ligand/receptor pairs of the TNF superfamily in VKH. In our dataset, the Th17 and Th1 cells shared increased TNF–FAS and TNFSF13 (BAFF) –FAS interaction with cDCs in VKH. Increased TNF and BAFF signaling are important factors orchestrating sustained inflammation in Th1 and Th17 cells^76^. The TNF–FAS signaling was also predicted to be activated in the interaction between cDCs and MAIT and in CD8TEM cells (Fig. 7c). The increased TNF signaling in CD8^+^ T cells resulted in the activation of NF-κB and MAPK cascades, which is in agreement with our previous findings (Fig. 4e). The TNF–ICOS interaction was only increased in the Treg cells, regulating Treg cell function. We noticed that CD40L/CD40 interactions were predicted to be increased in Th1, Th17, Treg, and MAIT cells. This has been reported to be a key regulatory interaction in autoimmune diseases that engages antigen-presenting cells and enhances proinflammatory cytokine production^77^. In our analysis, the cDCs were predicted to increase the chemokine pairs such as CCL3L1–DPP4, CCL3–CCR1, CCL3L1–CCR1, and CCL4L2–VSIR. These interactions, which limit the myeloid cell lineage and T helper cells, promote immune cell chemotaxis and migration (Fig. 7c). Altogether, our data suggest the potential of cDCs in regulating multiple immune cell subsets via cellular interactions, including TNF superfamily and chemokines. Further experiments are required to investigate whether cDCs are responsible for the regulation that might lead to the initiation of the inflammatory response in T cells and myeloid cell activation.

## Discussion

Our integrated single-cell multiomic analysis of PBMCs provides a comprehensive understanding of the cellular heterogeneity and cellular phenotypes underlying the pathogenesis of VKH. This analysis enabled us to (1) map the single-cell atlas of PBMCs in VKH and identify rare cell-specific TFs in human peripheral blood; (2) illustrate the pro-inflammatory role of NF-κB in VKH; (3) investigate the chromatin and transcriptional reprogramming in cDCs by integrative analysis of scRNA-seq and scATAC-seq datasets; (4) dissect the pathogenic activation of RELA in cDCs and reveal the link with prognosis in VKH; and (5) study the paired ligand-receptor between cDCs and lymphocytes, supporting the key role of cDCs in immune regulation in VKH patients.

Hu et al.^17^ have previously performed a single-cell RNA study on the VKH patients’ peripheral monocytes. Consistently, we also identified a group of pro-inflammatory monocytes (Supplementary Fig. 8c) that may be responsible for the induction of cytokine^17^. Further animal studies are needed to evaluate whether these proinflammatory monocytes are regulated by the TF activity of NF-κB family.

Epigenetic reprogramming is known to play an important role in the pathogenesis of autoimmune disease^44^. Imbalance of immune responses and overproduction of inflammatory cytokines in VKH disease has been associated with aberrant epigenetic changes^78–80^. Our profiling of patients with VKH disease allows us to demonstrate that T effector cell subsets have a highly activated phenotype, supports these findings^14–16^. For example, the activation of Th17 cells in VKH disease might lead to pathogenicity which might be driven by the activation of transcription factor T-bet.

Much attention has been focused on the role of cDCs in autoimmune diseases^47,81^. We identified distinct TF regulatory characteristics in cDCs in the VKH. Further analysis of the putative TF regulated network showed that high RELA activity in the cDCs was associated with poor prognosis. Intriguingly, *CCR7*^+^ *LAMP3*^+^ cDCs have been recently reported in cancer and have been characterized as mature DCs with a high potential for migration^82,83^. Lysosomal-associated membrane protein 3 (LAMP3)^+^ cDCs are also involved in the pathogenic cellular microenvironment, with resistance to anti-TNF therapy in Crohn’s disease^84^. A recent study utilized scRNA-seq with flow cytometry and low-input proteomics to identify cDCs as an important player in ocular cell infiltration in HLA-B27^+^ uveitis^85^. Our study suggests that NF-κB and its subunit might be important regulators of cDC activation and maturation. Further animal experiments are needed to confirm their regulatory effects on the antigen-presenting capacity of cDCs^86^. Consistently, enhanced NF-κB signaling in the cDCs has recently been described as a baseline predictive factor for patients non-responsive to anti-TNF therapy in psoriasis^87^. Importantly, we found that patients with higher RELA activity had a poorer prognosis than those with lower RELA activity. Further studies with long-term observations are required to confirm this finding.

In summary, cDCs might work as a key pro-inflammatory player and lymphocyte activator in VKH. Our single-cell multiomic atlas of human peripheral immune cells offers insights into the pathogenesis of VKH and its therapeutic options.

## Materials and methods

### Human subjects

This study was approved by The Ethics Committee of Zhongshan Ophthalmic Center (Guangzhou, China, 2019KYPJ114). All the participating individuals provided Written informed consent in the study. The relevant ethical regulations regarding human research participants were followed in accordance with the Declaration of Helsinki. All healthy individuals and patients were recruited from Zhongshan Ophthalmic Center. Individuals with comorbid conditions including cancer, immunocompromising disorders, hypertension, diabetes, and steroid use were excluded. The 12 healthy subjects (HC) consisted of 6 men and 6 women, with an average age of 39.9 years old. In the first VKH patient cohort (Supplementary Table 1), there were seven men and five women aged between 16 and 65 years. No significant differences in gender or age was detected between the HC and VKH groups. The diagnosis of VKH disease was based on the revised diagnostic criteria established by the First International Workshop on VKH Disease^88^. In the second VKH patient cohort, 89 VKH patients (38 men and 51 women) were recruited and followed up to determine whether they developed complications such as cataract, glaucoma, choroidal neovascularization, and subretinal fibrosis. During the 3-month follow-up, 35 patients (39.3%) developed at least one complication (Supplementary Data 2), and they were classified as those with a poor prognosis^5,7,8^.

### Cell isolation

To isolate PBMCs, all the peripheral venous blood samples were collected from healthy donors or patients using Ficoll-Hypaque density solution, heparinized, and then centrifuged for 30 min. Trypan blue staining was used to determine the viability and quantity of PBMCs in single-cell suspensions. For each sample, we ensured the cell viability exceeded 90% for the following experiment. For each sample with more than 1 × 10^7^ viable cells, a fraction of PBMCs was extracted for scRNA-seq analysis, and a fraction of PBMCs was allocated for single-cell assays for transposase-accessible chromatin sequencing (scATAC-seq).

### scATAC-seq processing

The nuclei isolation, washing and counting of nuclei suspensions were performed according to the manufacturer’s protocol. Based on the number of cells and desired final nuclei concentration, an appropriate volume of chilled Diluted Nuclei Buffer (10x Genomics; PN-2000153) was used to resuspend nuclei. The resulting nuclei concentration was determined using a Countess II FL Automated Cell Counter. Nuclei were then immediately used to generate 10× single cell ATAC libraries in Berry Genomics Co., Ltd. (Beijing, China). Libraries were uniquely barcoded and quantified using RT-qPCR. Each sample library was loaded on an Illumina Novaseq 6000 with 3.5 pmol/L loading concentration after pooling in pair-end mode. Next, Libraries were sequenced to either 90% saturation or 30,000 unique reads per cell on average. We followed the protocols for sample processing, library preparation, and instrument and sequencing settings on the 10× Chromium platform at https://support.10xgenomics.com/single-cell-atac. Raw sequencing data were converted to fastq format using Cellranger atac mkfastq (10× Genomics, v.1.0.0). scATAC-seq data reads were aligned to the GRCh38 (hg38) reference genome and quantified using the Cellranger count function (10x Genomics, v.1.0.0).

### scATAC-seq quality control

Arrow files were generated using ArchR v0.9.5^20^ by reading in accessible read fragments for each sample, following the default augments, unless otherwise indicated. To make sure that each cell had a high signal and well-sequenced, we filtered cells with less than 2500 unique fragments and enrichment at TSSs below 9. Doublets were inferred and filtered using ArchR^20^. We also removed the cells that mapped into blacklist regions based on the ENCODE project reference.

### scATAC-seq dimensionality reduction and clustering

We performed a layered dimensionality reduction approach using latent semantic indexing (LSI) and singular value decomposition (SVD), followed by Harmony^22^ batch correction based on each sample. Subsequently, single-cell accessibility profiles were clustered using Seurat’s shared nearest neighbor (SNN)^21^ graph clustering with ‘FindClusters’ at a default resolution of 0.8 on the harmonized LSI dimensions. During the reclustering step, clustering with ‘FindClusters’ at a default resolution of 0.3–1.5 to better identify small clusters. All data were visualized using uniform manifold approximation and projection (UMAP) in two-dimensional space.

### scATAC–seq gene activity scores

Gene activity scores were calculated based on the accessibility within the gene body, at the promoter and at distal regulatory elements was correlated with gene expression using ArchR v.0.9.527 with default parameters^20^. We also used additionally imputed weight method MAGIC ^89^on the resulting gene activity scores for reducing noise of the scATAC-seq data sparsity.

### scATAC–seq pseudobulk replicate generation and peak calling

For differential comparisons of clusters, cell types, and clinical states, non-overlapping pseudobulk replicates were generated from groups of cells using the ‘addGroupCoverages’ function with different arguments. These pseudobulk replicates were then used to generate the peak matrix (using ‘addReproduciblePeakSet’). We further used MACS2^90^ to perform peak calling. The pseudobulk peak set was used for downstream analysis.

### scATAC motif enrichment and motif deviation analysis

We performed motif enrichment and motif deviation analyses on the pseudobulk peak set. We used the Catalog of Inferred Sequence Binding Preferences (CIS-BP) motif (from ChromVAR)^40^, JASPAR2020 motif^91^ and HOMER^65^ to perform peak annotation. Additionally, the chromVAR deviation scores for these motifs were computed using ArchR implementation.

### scATAC–seq differential analysis

The pseudobulked peak set was used for differential analysis between different cell types and different clinical states using the ‘getMarkerFeatures’ function. We defined peak intensity as log_2_ of the normalized read counts. We used Wilcoxon test and Benjamini-Hochberg multiple test to calculate the *p* value and FDR between any pair of samples. Differentially accessible distal peaks were defined as FDR ≤ 0.1 and log2-fold change ≥0.5^92^.

### scATAC–seq Gene Ontology annotation and genomic regions annotation

In the differential analysis, we used the “annotatePeak” function in the ChIPseeker package^23^ to annotate the nearest genes in the peak region with default arguments. Subsequently, we used the nearest genes as in the Metascape webtool (www.metascape.org)^93^ which allows visualization of functional patterns of gene clusters. Statistical analyses were performed to conduct DEG gene ontology and pathway enrichment. A *p* value of less than 0.05 was considered statistically significant.

### scATAC–seq TF Foot-print analysis

Motif footprint analysis was performed by measuring Tn5 insertions in genome-wide motifs and normalized by subtracting the Tn5 bias from the footprinting signal. For each peak set, we used CIS-BP motifs (from chromVAR motifs human_pwms_v1)^40^ or JASPAR2020 motifs^40^ to calculate motif positions. We normalized these footprints using mean values ±200–250 from the motif center. We then plotted the mean and standard deviation for each footprint pseudo-replicate.

### scATAC–seq ChromVAR deviation enrichment of human eye and skin tissues

In ChromVAR deviation enrichment^40^, we downloaded the bulk ATAC-seq and CHIP-seq data of the healthy human retina, macular, retina pigment epithelial, and skin^46,47^. The.bw files were read and processed using the Rtracklayer package^94^. We identified the cis-elements in each tissue and extended them ±2.5 kb. A ‘GRangesList’ object was created with a feature set of peaks for downstream analysis. Next, we used the pipeline designed by Satpathy et al.^19^ to calculate the co-accessibility in our scATAC-seq dataset for each single-cell group using Cicero^95^ and created a connection matrix (Supplementary Fig. 6f). To identify co-accessible peaks in each tissue within our scATAC-seq data, we then overlapped the peaks with the connection matrix. We kept the matrix with peaks that over 3 co-accessibility (Supplementary Fig. 6f). To computed the GC bias-corrected deviations, we used the chromVAR “computeDeviations” and “computeVariability” function with default parameters (Supplementary Fig. 6f).

### scATAC-seq peak to gene linkage analysis

To identify peak-to-gene links prediction, we used the ArchR ‘addPeak2GeneLinks’ function and set the parameter ‘corCutOff’ as 0.2, ‘reducedDims’ as the dimensionality reduction results after batch corrected. The returned ‘GRanges’ object were used for visualization.

### scATAC–seq GWAS SNPs liftover and DARs mapping

We downloaded the GWAS data from GWAS Catalog (https://www.ebi.ac.uk/gwas/) using the searching term ‘Vogt- Koyanagi-Harada disease’. The collected GWAS data was identified by Hou et al.^45^. All the gene locus of the SNPs was chosen for inferring peak-to-gene linkages. To pinpoint the GWAS SNPs to our datasets, we used the UCSC utility liftOver (https://genome.ucsc.edu/cgi-bin/hgLiftOver) to lift the GWAS SNPs from hg19 to hg38. We then took the set of differentially accessible peaks (in the positive direction) for each cell type and annotated each SNP according to whether it overlapped one of these peaks. Only the locus with over 0.2 correlations between SNPs and genes were kept.

### scRNA-seq processing

The scRNA-seq libraries were barcoded and converted using The Chromium Single Cell 5 Library (the 10× Genomics chromium platform), Gel Bead and Multiplex Kit, and Chip Kit (10× Genomics). According to the manufacturer’s protocols, we prepared the Single-cell RNA libraries using the Chromium Single Cell 5*'* v2 Reagent (10x Genomics, 120237) kit. The libraries for scRNA-seq experiments were sequenced on Illumina NovaSeq6000 in pair-end mode. The quality of the libraries was checked using the FastQC software. The sequenced data were first processed and aligned to the GRCh38 reference for each sample using CellRanger software with the default parameter (https://support.10xgenomics.com, version 3.1.0).

The Cell Ranger-count function in CellRanger Software Suite (10x Genomics) was used to demultiplex and barcode the sequences derived from the 10x Genomics single-cell RNA-seq platform. The data were filtered, normalized, and dimensionality was reduced and clustered. We then used CellRanger-aggr to aggregate all the samples for downstream analysis.

### scRNA-seq quality control

For quality control, cells were filtered out with more than 11% of mitochondrial genes and fewer than 200 or more than 3000 detected genes using Seurat V3^21^. We further filtered the cell populations identified as red blood cells and platelets that expressed *HBB*, *HBA1*, *PPBP*, and *PF4* genes.

### scRNA-seq dimensionality reduction and clustering

After normalization, scale data with the top 5000 most variable genes using the ‘FindVariableFeatures’ function in R package Seurat v3. We performed principal component analysis using variable genes, and the first 30 principal components (PCs) were further used to deal with batch effect issues using the Harmony package based on each sample. We then performed Seurat clustering on the Harmony to batch-correct dimensions at the resolution of 0.8. We further performed the UMAP analysis, a dimensionality-reducing visualization tool, was used to embed the dataset into two dimensions.

### scRNA–seq differential analysis

For scRNA-seq differential expression analysis, we used the “FindAllMarkers” function of the Seurat package with default parameters. A *p* value of less than 0.05 was considered statistically significant^96^.

### scRNA-seq signature score analysis

To assess the inflammatory state in circulating dendritic cells, we collected all marker genes from inflammatory *CD1c*^+^ dendritic cells^72^. Inflammatory signature scores were estimated for all cells as the average of the scaled Z-normalized expression of the genes in the list. The scores were calculated as follows: the score of the gene set in the given cell subset (named as X) was computed as the sum of all UMI for all the genes expressed in X cells, divided by the sum of all UMI expressed by X cell^38,97^.

### Multiomics data processing

To integrate scRNA-seq and scATAC-seq dataset, we followed the integration pipeline described in ArchR, Seurat and Signac^98^ website. First, we implemented the ArchR built-in method to divide the total dataset into smaller groups of cells and performed separate alignments for saving computational RAM. We then applied Seurat’s canonical correlation analysis (CCA) to integrate our epigenetic and transcriptomic data. No further batch correction method was used. For this purpose, the integration analysis was based on the log-normalized and scaled scATAC-seq gene score matrix with the scRNA-seq gene expression matrix. By directly aligning cells from scATAC-seq with cells from scRNA-seq, the union of the 2000 most variable genes was used in each modality as input to Seurat’s “FindTransferAnchors” function and Seurat’s “TransferData” function with “weight.reduction” set to the dimensionality of scATAC-seq dataset after Harmony batch correction while other parameters were set to default. For each cell profiled by scRNA-seq and each cell profiled by scATAC-seq, we identified the nearest neighbor cell in the respective other modality by applying a nearest-neighbor search in the joint space CCA L2 space. These nearest-neighbor-based cell matches from all gestational time points were concatenated to obtain dataset wide cell matches across both modalities.

### Pseudotime analysis

To order cells in pseudotime, we identified a trajectory and then aligned single cells across the trajectory in scATAC-seq dataset, scRNA-seq dataset as well as the merged dataset^42^. Based on the user-defined trajectory backbone, cellular trajectories were established in a low-dimensional space using batch-corrected LSI embeddings. CD14^+^ monocyte subsets were provided to ArchR^20^ using ‘addTrajectory’ function with “preFilterQuantile” and “postFilterQuantile” set to 0.95 while other parameters were set to default. Then, a k-nearest neighbor algorithm was used to order cells based on the Euclidean distance of each cell to the nearest cluster’s centroid. Cells were then assigned pseudotime value estimates, and a heatmap was plotted using differential feature z-scores that were associated with the pseudotime trajectory.

### Identifying TF target genes

To identify significantly shared TFs and their directly regulated target genes in VKH disease, we used the framework designed by Granja et al.^73^. We first identified a set of TFs whose hypergeometric enrichment in differential peaks between VKH patients and healthy subjects, and the enrichment was identified as being transcriptionally correlated with the accessibility of their motifs (see above). Next, for a given TF and all identified peak-to-gene links, we further subset these links by those containing the TF motif. For each peak-to-gene link, we determined whether both the peak and the gene were upregulated in the VKH group. In addition, for each gene that has at least one differential peak-to-gene link, we summed their squared correlation and defined that as the differential linkage score.

### Receptor–ligand pair analysis

Receptor–ligand analysis between cDCs and other immune cell subpopulations was performed using CellphoneDB statistical analysis, v.2.0^75^. We extracted the gene matrix from scRNA-seq data between different clinical state groups to perform this analysis. We selected the ligand/receptor interactions with more significant (*p* < 0.05) cell-cell interaction pairs in disease states than in healthy groups.

### RNA-seq library preparation, sequencing, and analysis

Total RNA was extracted from the blood samples following the manufacturer’s instructions. The libraries were sequenced using an MGI-2000 sequencing instrument. The quality control process included adapter trimming and low-quality read removal using Trim Galore (v0.6.4; https://github.com/FelixKrueger/TrimGalore) with parameters ‘—q 20 –phred 33 –stringency 3 –length 20 –e 0.1’. The clean mRNA data were mapped to the human genome GRCh38 using Bowtie2^99^ (v2.3.5.1; http://bowtie-bio.sourceforge.net/bowtie2/index.shtml). Bam files were then sorted using SAMtools^100^ (v1.7; http://samtools.sourceforge.net/index.shtml). Gene counts and gene FPKM from bam files were then generated using ‘featureCounts’^101^.

### Survival analysis

For survival analysis, we matched FPKM gene expression to each sample ID. We computed row-wise z-scores for all genes that were identified as target genes for NFKB1 (n = 347) and RELA (n = 382). Next, we used the column means of this matrix to obtain an average z-score across all NKFB1 and RELA target genes. We further identified donors based on this expression. We computed *p* values using R package survival. Kaplan–Meier curve was plotted using the R package survminer ‘ggsurvplot’ in R.

### Statistics and reproducibility

Statistical analysis of the frequencies of immune cell subpopulations between groups was performed using one-way ANOVA tests with Bonferroni’s post-hoc correction with GraphPad Prism 8.0. Two-sided *p* values of less than 0.05, were considered statistically significant. All the statistical details for the experiments can be found in the figure legends as well as in the Method Details section. When comparing the gene expression levels between groups, we estimated the *p* values using the two-sided Wilcoxon test in R package ggpubr with default parameters. In estimating the GO biological process and pathway, *p* values were derived by a hypergeometric test with the default parameters in the Metascape webtool. Each figure legends include the details of the size of biological replicates and the assays.

### Reporting summary

Further information on research design is available in the Nature Research Reporting Summary linked to this article.

## Supplementary information

Supplementary Material

Description of Additional Supplementary Files

Supplementary Data 1

Supplementary Data 2

Reporting summary

## Data Availability

The scRNA-seq, scATAC-seq and bulk RNA-seq data analyzed in the article are available from the corresponding author upon request under the Project Accession No. PRJCA004696 and GSA Accession No. HRA001643 (Beijing Institute of Genomics).

## Code availability

The data analysis pipeline in our study was described on the ArchR websites and Seurat websites. The code for TF network analysis in this study can be found on Github at https://github.com/GreenleafLab/MPAL-Single-Cell-2019. The code for plotting in this study can be found on https://gitlab.com/cvejic-group/integrative-scrnascatac-human-foetal^42^.
