# Supplementary material for: Chromatin accessibility analysis reveals regulatory dynamics and therapeutic relevance of Vogt-Koyanagi-Harada disease
Source: Commun Biol. 2022 May 26;5:506. doi: 10.1038/s42003-022-03430-9 (PMC9135711; doi:10.1038/s42003-022-03430-9)
Supplement: Supplementary file 1 — Supplementary Material [file 42003_2022_3430_MOESM1_ESM.pdf]

Supplementary Information

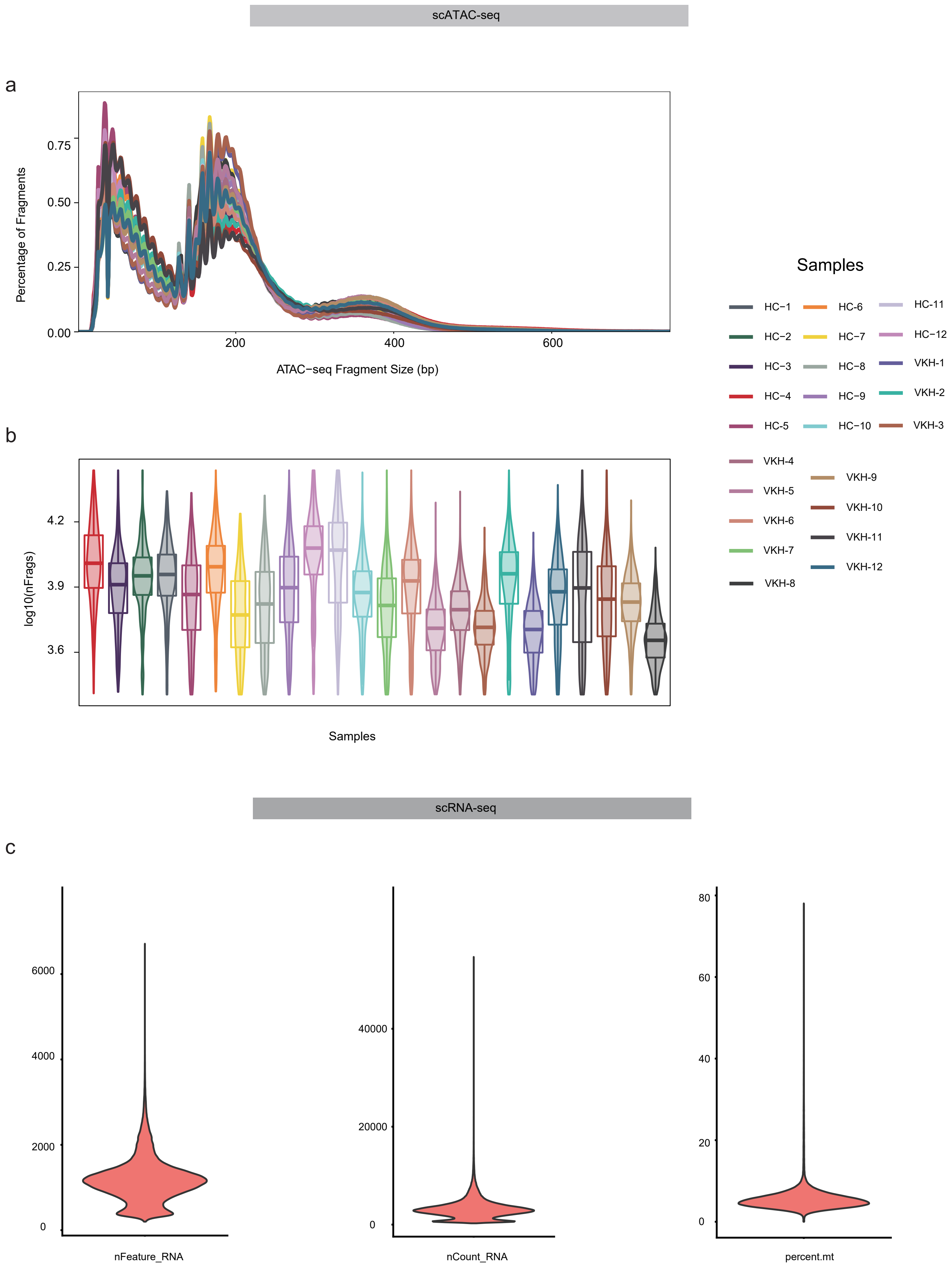
scATAC-seq
a
Percentage of Fragments
0.00
0.25
0.50
0.75
200
400
600
ATAC−seq Fragment Size (bp)
Samples
HC−1
HC−2
HC−3
HC−4
HC-5
HC-6
HC−7
HC−8
HC−9
HC−10
HC-11
HC−12
VKH-1
VKH-2
VKH-3
VKH-4
VKH-5
VKH-6
VKH-7
VKH-8
VKH-9
VKH-10
VKH-11
VKH-12
b
log10(nFrags)
3.6
3.9
4.2
Samples
scRNA-seq
c
0
2000
4000
6000
nFeature_RNA
0
20000
40000
nCount_RNA
0
20
40
60
80
percent.mt

Supplementary Figure 1. Quality control of scATAC-seq and scRNA-seq data.

a Aggregated scATAC-seq fragment size distributions across individual experiments demonstrating sub-, mono- and multi nucleosome spanning ATAC-seq fragments. **b** Violin plot of the scATAC-seq fragment size distributions passing filter for each experimental sample. **c** Violin plot of the features, counts, and percentage of mitochondrial genes in scRNA-seq dataset. All data are aligned and annotated to hg38 reference genome.

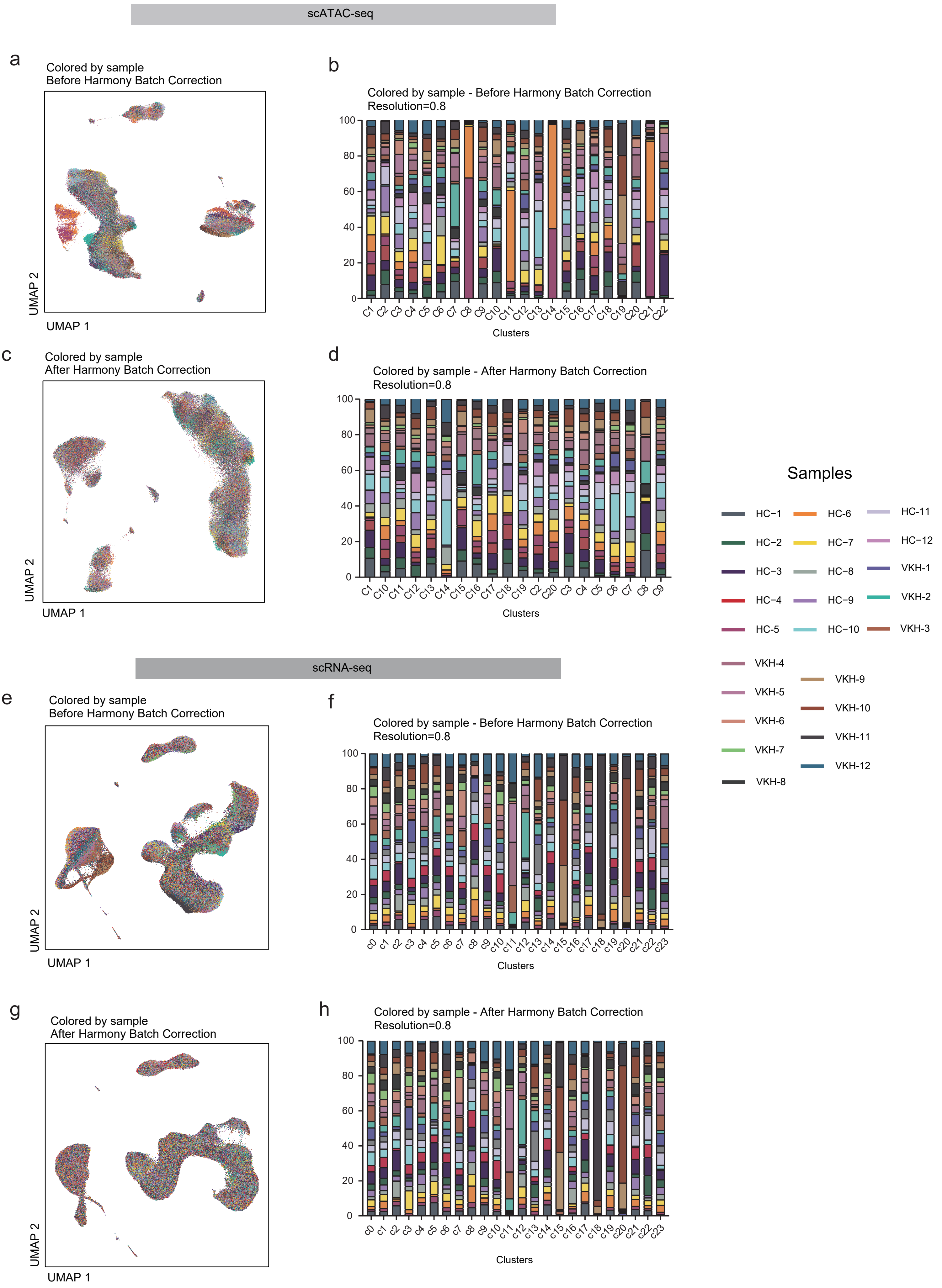

scATAC-seq
a
Colored by sample
Before Harmony Batch Correction
UMAP 2
UMAP 1
b
Colored by sample - Before Harmony Batch Correction
Resolution=0.8
100
80
60
40
20
0
C1 C2 C3 C4 C5 C6 C7 C8 C9 C10 C11 C12 C13 C14 C15 C16 C17 C18 C19 C20 C21 C22
Clusters
c
Colored by sample
After Harmony Batch Correction
UMAP 2
UMAP 1
d
Colored by sample - After Harmony Batch Correction
Resolution=0.8
100
80
60
40
20
0
C1 C10 C11 C12 C13 C14 C15 C16 C17 C18 C19 C2 C20 C3 C4 C5 C6 C7 C8 C9
Clusters
Samples
HC−1
HC−2
HC−3
HC−4
HC−5
HC-6
HC−7
HC−8
HC−9
HC−10
HC-11
HC−12
VKH-1
VKH-2
VKH-3
VKH-4
VKH-5
VKH-6
VKH-7
VKH-8
VKH-9
VKH-10
VKH-11
VKH-12
scRNA-seq
e
Colored by sample
Before Harmony Batch Correction
UMAP 2
UMAP 1
f
Colored by sample - Before Harmony Batch Correction
Resolution=0.8
100
80
60
40
20
0
c0 c1 c2 c3 c4 c5 c6 c7 c8 c9 c10 c11 c12 c13 c14 c15 c16 c17 c18 c19 c20 c21 c22 c23
Clusters
g
Colored by sample
After Harmony Batch Correction
UMAP 2
UMAP 1
h
Colored by sample - After Harmony Batch Correction
Resolution=0.8
100
80
60
40
20
0
c0 c1 c2 c3 c4 c5 c6 c7 c8 c9 c10 c11 c12 c13 c14 c15 c16 c17 c18 c19 c20 c21 c22 c23
Clusters

Supplementary Figure 2. Batch correction of scATAC-seq and scRNA-seq.

a scATAC-seq UAMP before harmony-based batch correction, colored by each sample (n= 24). **b** Cluster-specific composition in scATAC-seq dataset before harmony-based batch correction, colored by each sample (n= 24). **c** scATAC-seq UAMP after harmony-based batch correction, colored by each sample (n= 24). **d** Cluster-specific composition in scATAC-seq dataset after harmony-based batch correction, colored by each sample (n= 24). **e** scRNA-seq UAMP before harmony-based batch correction, colored by each sample (n= 24). **f** Cluster-specific composition in scRNA-seq dataset before harmony-based batch correction, colored by each sample (n= 24). **g** scRNA-seq UAMP after harmony-based batch correction, colored by each sample (n= 24). **h** Cluster-specific composition in scRNA-seq dataset after harmony-based batch correction, colored by each sample (n= 24). All data are aligned and annotated to hg38 reference genome.

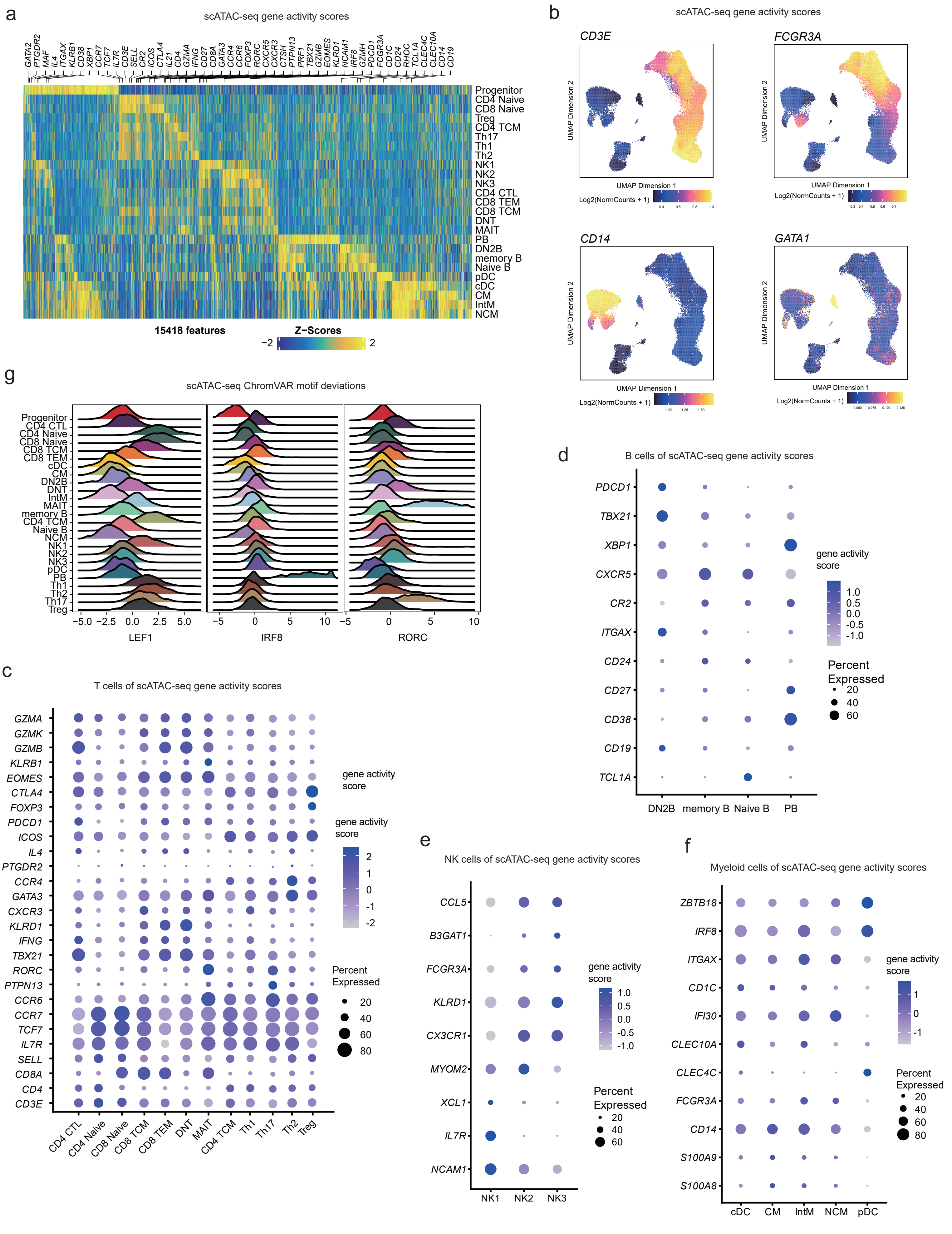
a
scATAC-seq gene activity scores
GATA2 PTGDR2 MAF IL4 ITGAX KLRB1 CD38 XBP1 CCR7 TCF7 IL7R CD3E SELL CR2 ICOS CTLA4 IL21 CD4 GZMA IFNG CD27 CD8A GATA3 CCR4 CCR6 FOXP3 RORC CXCR5 CXCR3 CTSH PTPN13 PRF1 TBX21 GZMB EOMES KLRD1 NCAM1 IRF8 GZMH PDCD1 FCGR3A CD1C CD24 RHOC TCL1A CLEC4C CLEC10A CD14 CD19
Progenitor
CD4 Naive
CD8 Naive
Treg
CD4 TCM
Th17
Th1
Th2
NK1
NK2
NK3
CD4 CTL
CD8 TEM
CD8 TCM
DNT
MAIT
PB
DN2B
memory B
Naive B
pDC
cDC
CM
IntM
NCM
15418 features
Z-Scores
−2
2
b
scATAC-seq gene activity scores
CD3E
FCGR3A
CD14
GATA1
UMAP Dimension 1
UMAP Dimension 2
Log2(NormCounts + 1)
g
scATAC-seq ChromVAR motif deviations
Progenitor
CD4 CTL
CD4 Naive
CD8 Naive
CD8 TCM
CD8 TEM
cDC
CM
DN2B
DNT
IntM
MAIT
memory B
CD4 TCM
Naive B
NCM
NK1
NK2
NK3
pDC
PB
Th1
Th2
Th17
Treg
−5.0 −2.5 0.0 2.5 5.0
LEF1
−5 0 5 10
IRF8
−5 0 5 10
RORC
d
B cells of scATAC-seq gene activity scores
PDCD1
TBX21
XBP1
CXCR5
CR2
ITGAX
CD24
CD27
CD38
CD19
TCL1A
DN2B
memory B
Naive B
PB
gene activity score
1.0
0.5
0.0
-0.5
-1.0
Percent Expressed
20
40
60
c
T cells of scATAC-seq gene activity scores
GZMA
GZMK
GZMB
KLRB1
EOMES
CTLA4
FOXP3
PDCD1
ICOS
IL4
PTGDR2
CCR4
GATA3
CXCR3
KLRD1
IFNG
TBX21
RORC
PTPN13
CCR6
CCR7
TCF7
IL7R
SELL
CD8A
CD4
CD3E
CD4 CTL
CD4 Naive
CD8 Naive
CD8 TCM
CD8 TEM
DNT
MAIT
CD4 TCM
Th1
Th17
Th2
Treg
gene activity score
gene activity score
2
1
0
-1
-2
Percent Expressed
20
40
60
80
e
NK cells of scATAC-seq gene activity scores
CCL5
B3GAT1
FCGR3A
KLRD1
CX3CR1
MYOM2
XCL1
IL7R
NCAM1
NK1
NK2
NK3
gene activity score
1.0
0.5
0.0
-0.5
-1.0
Percent Expressed
20
40
60
f
Myeloid cells of scATAC-seq gene activity scores
ZBTB18
IRF8
ITGAX
CD1C
IFI30
CLEC10A
CLEC4C
FCGR3A
CD14
S100A9
S100A8
cDC
CM
IntM
NCM
pDC
gene activity score
1
0
-1
Percent Expressed
20
40
60
80

Supplementary Figure 3. Validation of key marker genes for scATAC-seq dataset in HC group.

a Heatmap visualization of log-normalized gene activity scores of subpopulation-specific genes in peripheral blood. Selected genes are indicated. **b** UMAP projection colored by gene activity scores for the annotated lineage-defining genes in HC group of scATAC-seq dataset. The minimum and maximum gene activity scores are shown in each panel. **c** Dot plot of gene activity scores of the marker genes in T cell subsets. The dot size indicates the percentage of the cells in each cluster in which the gene of interest. The standardized gene activity score level is indicated by color intensity. **d** Dot plot of gene activity scores of the marker genes in B cell subsets. The dot size indicates the percentage of the cells in each cluster in which the gene of interest. The standardized gene activity score level is indicated by color intensity. **e** Dot plot of gene activity scores of the marker genes in NK cell subsets. The dot size indicates the percentage of the cells in each cluster in which the gene of interest. The standardized gene activity score level is indicated by color intensity. **f** Dot plot of gene activity scores of the marker genes in Myeloid cell subsets. The dot size indicates the percentage of the cells in each cluster in which the gene of interest. The standardized gene activity score level is indicated by color intensity. **g** Ridge plot of chromVAR bias-corrected deviations of indicated TFs across all healthy immune cell types. All data are aligned and annotated to hg38 reference genome.

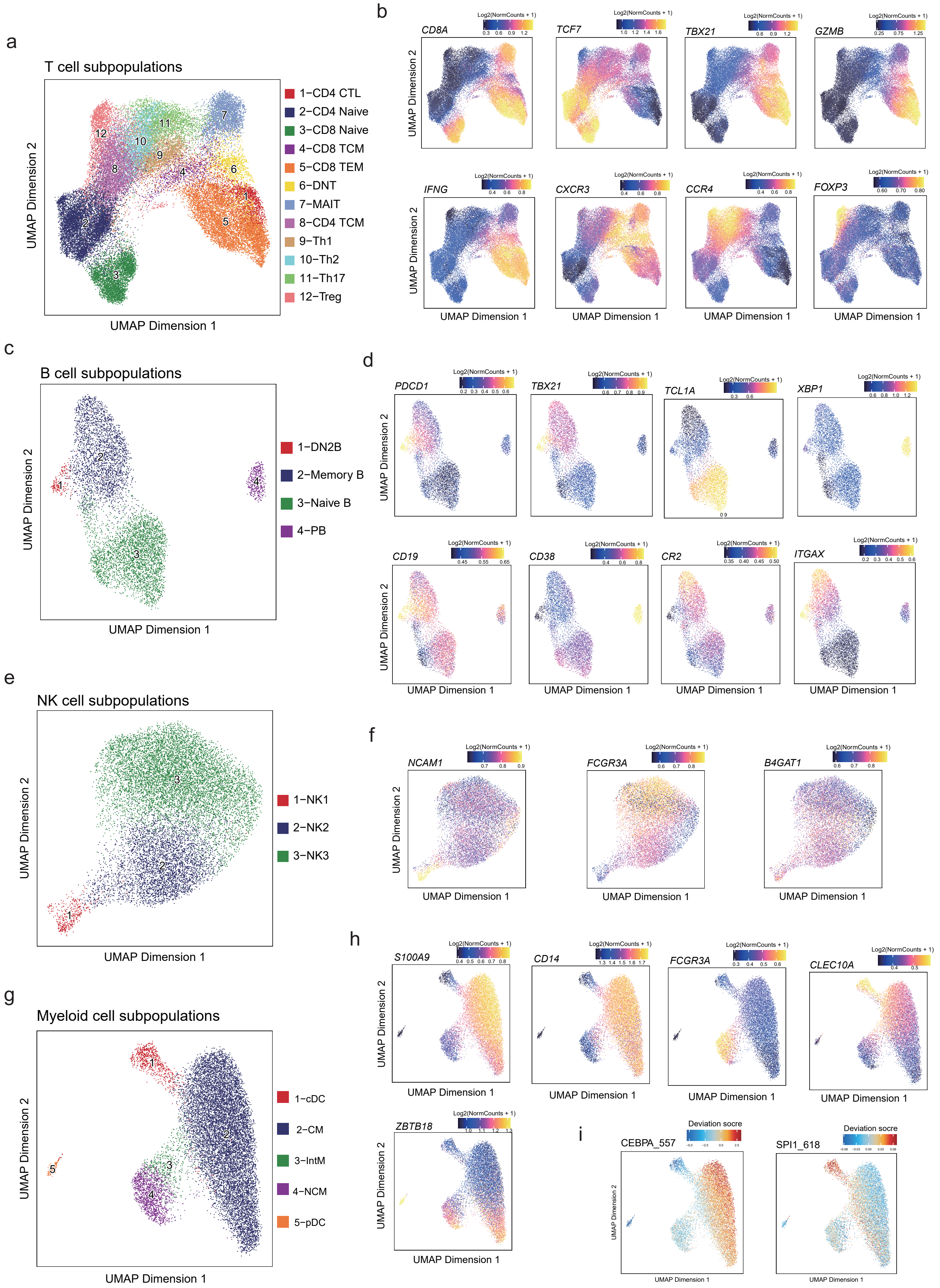
a
T cell subpopulations
1−CD4 CTL
2−CD4 Naive
3−CD8 Naive
4−CD8 TCM
5−CD8 TEM
6−DNT
7−MAIT
8−CD4 TCM
9−Th1
10−Th2
11−Th17
12−Treg
b
CD8A
TCF7
TBX21
GZMB
IFNG
CXCR3
CCR4
FOXP3
c
B cell subpopulations
1−DN2B
2−Memory B
3−Naive B
4−PB
d
PDCD1
TBX21
TCL1A
XBP1
CD19
CD38
CR2
ITGAX
e
NK cell subpopulations
1−NK1
2−NK2
3−NK3
f
NCAM1
FCGR3A
B4GAT1
g
Myeloid cell subpopulations
1−cDC
2−CM
3−IntM
4−NCM
5−pDC
h
S100A9
CD14
FCGR3A
CLEC10A
ZBTB18
i
CEBPA_557
SPI1_618
Deviation socre
Log2(NormCounts + 1)
UMAP Dimension 1
UMAP Dimension 2

Supplementary Figure 4. Validation of key marker genes for scATAC-seq subgroup analysis in HC group.

a UMAP projection of T cell subgroup profiles of peripheral blood immune cell types from 12 HCs. Dots represent individual cells, and colors indicate T cell subpopulation cell type. **b** UMAP projection of T cell subgroup profiles colored by gene activity scores for the annotated lineage-defining genes in HC group of scATAC-seq dataset. The minimum and maximum gene activity scores are shown in each panel. **c** UMAP projection of B cell subgroup profiles of peripheral blood immune cell types from 12 HCs. Dots represent individual cells, and colors indicate T cell subpopulation cell type. **d** UMAP projection of B cell subgroup profiles colored by gene activity scores for the annotated lineage-defining genes in HC group of scATAC-seq dataset. The minimum and maximum gene activity scores are shown in each panel. **e** UMAP projection of NK cell subgroup profiles of peripheral blood immune cell types from 12 HCs. Dots represent individual cells, and colors indicate T cell subpopulation cell type. **f** UMAP projection of NK cell subgroup profiles colored by gene activity scores for the annotated lineage-defining genes in HC group of scATAC-seq dataset. The minimum and maximum gene activity scores are shown in each panel. **g** UMAP projection of Myeloid cell subgroup profiles of peripheral blood immune cell types from 12 HCs. Dots represent individual cells, and colors indicate T cell subpopulation cell type. **h** UMAP projection of Myeloid cell subgroup profiles colored by gene activity scores for the annotated lineage-defining genes in HC group of scATAC-seq dataset. The minimum and maximum gene activity scores are shown in each panel. **i** UMAP projection of Myeloid cell subgroup of scATAC-seq peripheral blood profiles colored by chromVAR TF motif bias-corrected deviations for the indicated factors. All data are aligned and annotated to hg38 reference genome.

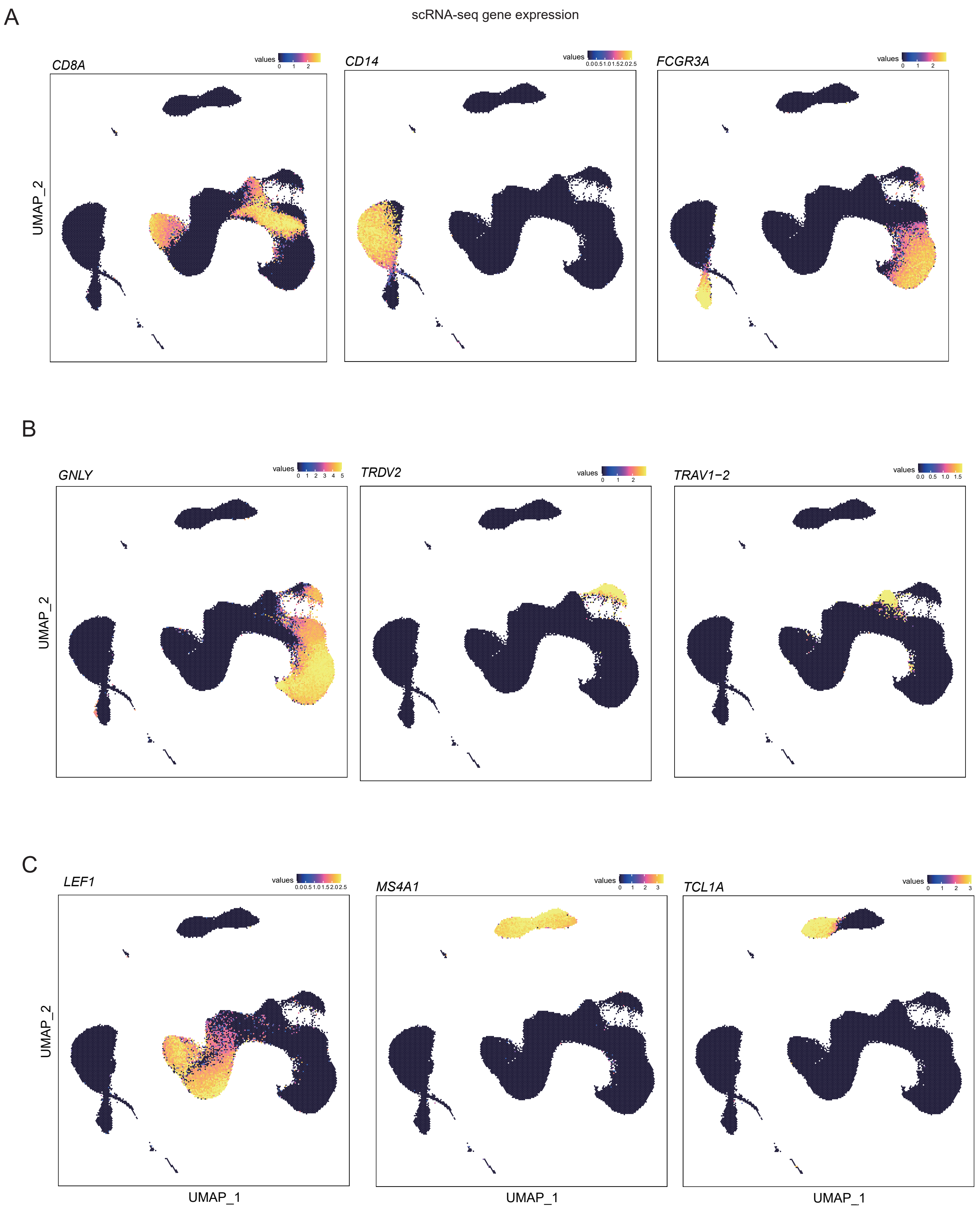

A
scRNA-seq gene expression
CD8A
CD14
FCGR3A
B
GNLY
TRDV2
TRAV1−2
C
LEF1
MS4A1
TCL1A
values
UMAP_2
UMAP_1

Supplementary Figure 5. Validation of key marker genes for scRNA-seq dataset.

a UMAP projection colored by log-normalized gene expression to the indicated gene. **b** UMAP projection colored by log-normalized gene expression to the indicated gene. **c** UMAP projection colored by log-normalized gene expression to the indicated gene. All data are aligned and annotated to hg38 reference genome.

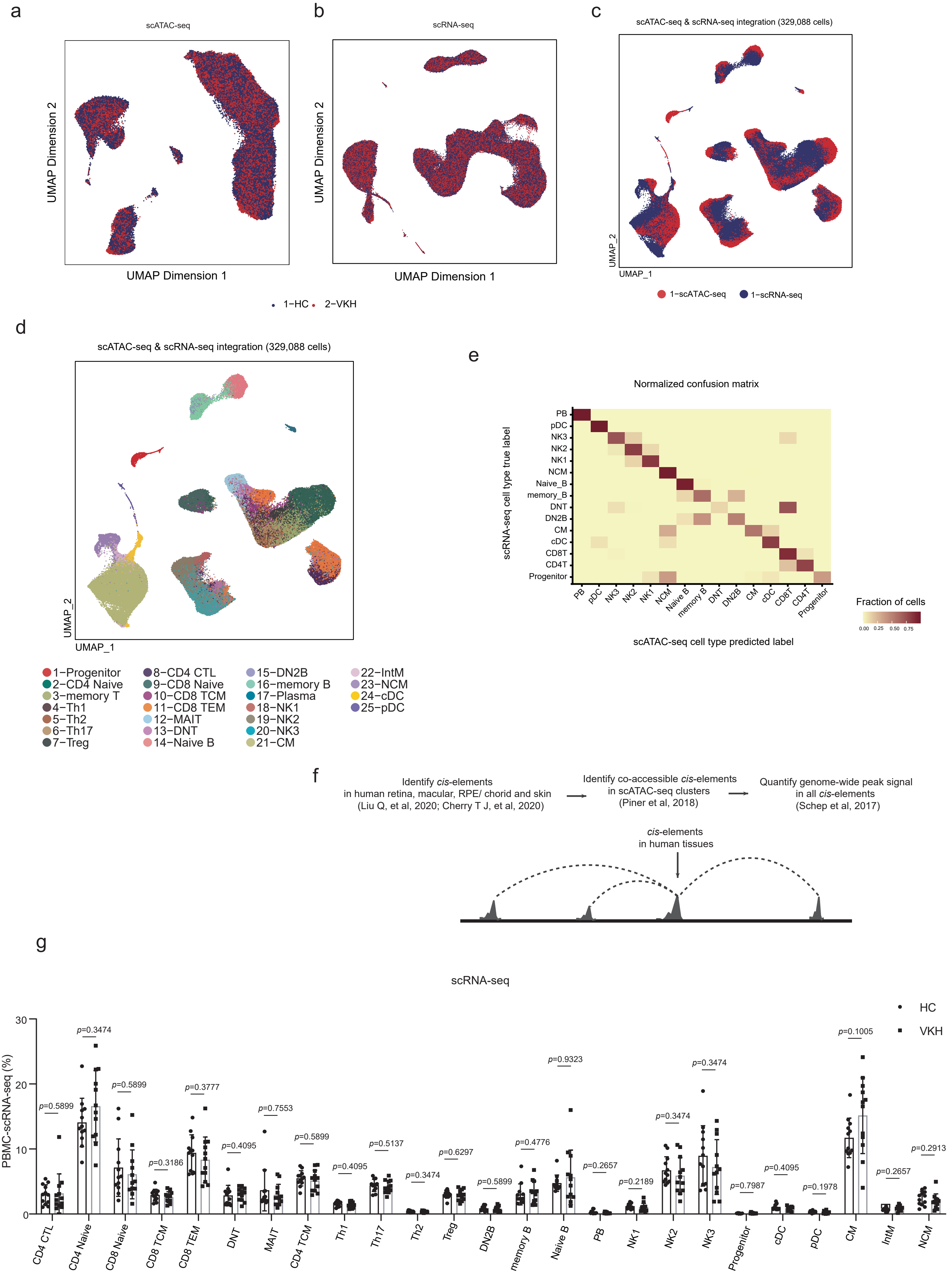

a
scATAC-seq
UMAP Dimension 2
UMAP Dimension 1
b
scRNA-seq
UMAP Dimension 2
UMAP Dimension 1
1−HC
2−VKH
c
scATAC-seq & scRNA-seq integration (329,088 cells)
UMAP_2
UMAP_1
1−scATAC-seq
1−scRNA-seq
d
scATAC-seq & scRNA-seq integration (329,088 cells)
UMAP_2
UMAP_1
1−Progenitor
2−CD4 Naive
3−memory T
4−Th1
5−Th2
6−Th17
7−Treg
8−CD4 CTL
9−CD8 Naive
10−CD8 TCM
11−CD8 TEM
12−MAIT
13−DNT
14−Naive B
15−DN2B
16−memory B
17−Plasma
18−NK1
19−NK2
20−NK3
21−CM
22−IntM
23−NCM
24−cDC
25−pDC
e
Normalized confusion matrix
scRNA-seq cell type true label
PB
pDC
NK3
NK2
NK1
NCM
Naive_B
memory_B
DNT
DN2B
CM
cDC
CD8T
CD4T
Progenitor
PB
pDC
NK3
NK2
NK1
NCM
Naive B
memory B
DNT
DN2B
CM
cDC
CD8T
CD4T
Progenitor
scATAC-seq cell type predicted label
Fraction of cells
0.00
0.25
0.50
0.75
f
Identify cis-elements
in human retina, macular, RPE/ chorid and skin
(Liu Q, et al, 2020; Cherry T J, et al, 2020)
Identify co-accessible cis-elements
in scATAC-seq clusters
(Piner et al, 2018)
Quantify genome-wide peak signal
in all cis-elements
(Schep et al, 2017)
cis-elements
in human tissues
g
scRNA-seq
PBMC-scRNA-seq (%)
30
20
10
0
HC
VKH
p=0.5899
p=0.3474
p=0.5899
p=0.3186
p=0.3777
p=0.4095
p=0.7553
p=0.5899
p=0.4095
p=0.5137
p=0.3474
p=0.6297
p=0.5899
p=0.4776
p=0.9323
p=0.2657
p=0.2189
p=0.3474
p=0.3474
p=0.7987
p=0.4095
p=0.1978
p=0.1005
p=0.2657
p=0.2913
CD4 CTL
CD4 Naive
CD8 Naive
CD8 TCM
CD8 TEM
DNT
MAIT
CD4 TCM
Th1
Th17
Th2
Treg
DN2B
memory B
Naive B
PB
NK1
NK2
NK3
Progenitor
cDC
pDC
CM
IntM
NCM

Supplementary Figure 6. Integration analysis of scATAC-seq and scRNA-seq dataset among HC and VKH.

a UMAP projection of scRNA-seq dataset colored by different clinical states (HC and VKH) as indicated. (nHC=88,429 cells, nVKH=107,519 cells). **b** UMAP projection of scATAC-seq dataset colored by different clinical states (HC and VKH) as indicated. (nHC=74,510 cells, nVKH=58,630 cells). **c** UMAP projection of integrated scATAC-seq and scRNA-seq dataset colored by different sequencing methods (scATAC-seq and scRNA-seq) as indicated (n= 329,088 cells). **d** UMAP projection of integrated scATAC-seq and scRNA-seq dataset colored by different cell types as indicated (n= 329,088 cells). **e** Confusion matrix showing the cell type assignment achieved by the Seurat's canonical correlation analysis. The color intensity represents the fraction of the assigned cells per cell type. **f** Statistical comparison of the frequencies of immune subpopulations in scRNA-seq dataset. Indicated *p* values are comparing VKH with HC. All *p* values were calculated using one-way ANOVA test. Controlling for multiple comparisons was performed with the Dunnett's multiple comparisons test. **g** Analysis workflow for chromVAR deviation enrichment analysis of human tissues using Cicero co-accessibility. All data are aligned and annotated to hg38 reference genome.

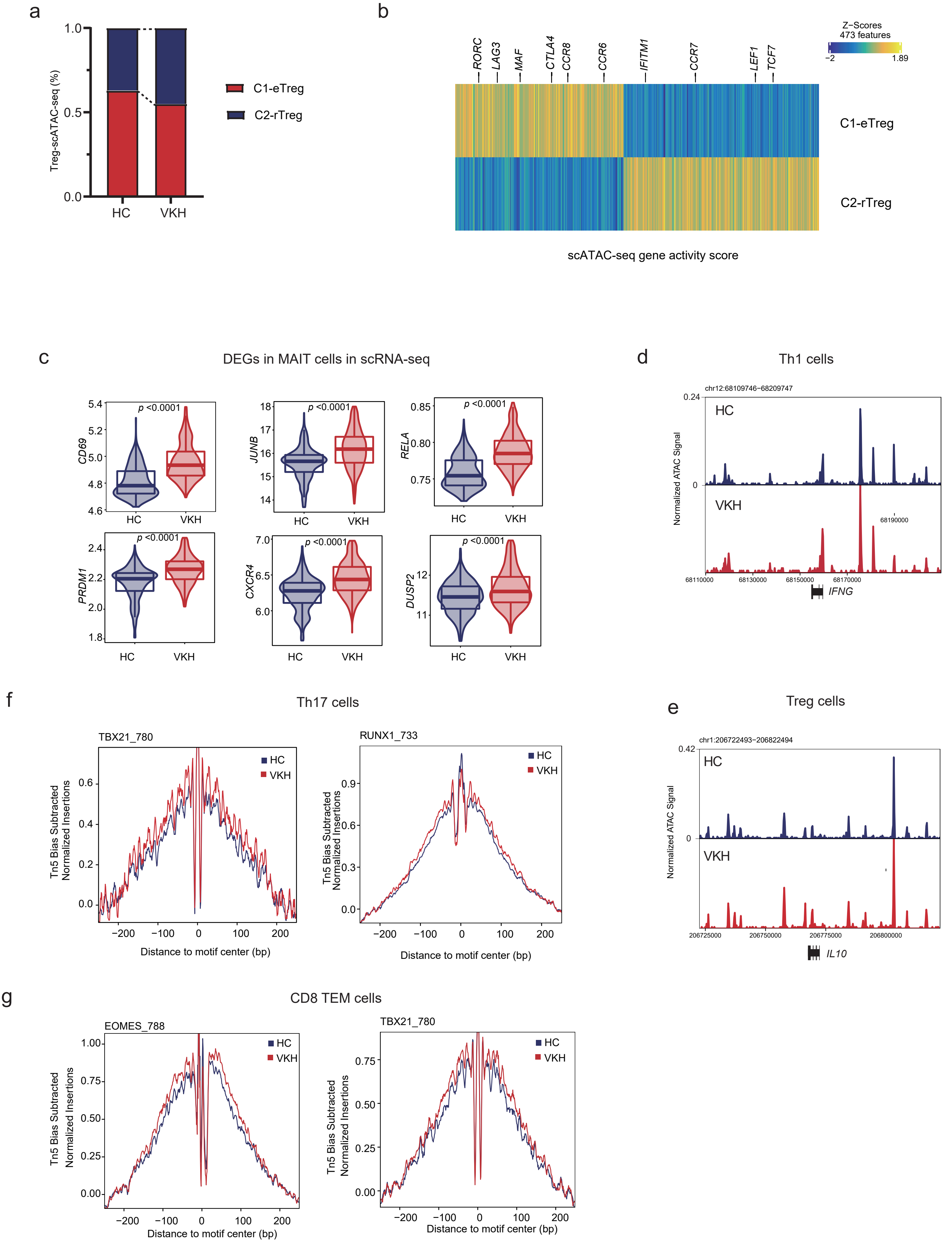
a
Treg-scATAC-seq (%)
1.0
0.5
0.0
HC
VKH
C1-eTreg
C2-rTreg
b
Z–Scores
473 features
−2
1.89
RORC
LAG3
MAF
CTLA4
CCR8
CCR6
IFITM1
CCR7
LEF1
TCF7
C1-eTreg
C2-rTreg
scATAC-seq gene activity score
c
DEGs in MAIT cells in scRNA-seq
p <0.0001
CD69
JUNB
RELA
PRDM1
CXCR4
DUSP2
HC
VKH
d
Th1 cells
chr12:68109746−68209747
0.24
Normalized ATAC Signal
68190000
68110000
68130000
68150000
68170000
IFNG
e
Treg cells
chr1:206722493−206822494
0.42
206725000
206750000
206775000
206800000
IL10
f
Th17 cells
TBX21_780
RUNX1_733
Tn5 Bias Subtracted
Normalized Insertions
Distance to motif center (bp)
g
CD8 TEM cells
EOMES_788
TBX21_780

Supplementary Figure 7. Changes in T cell subsets of scATAC-seq and scRNA-seq dataset among HC and VKH.

a Relative cluster abundance of Treg in HC (n=12) and VKH groups (n=12) in scATAC-seq dataset. **b** Heatmap visualization of log-normalized gene activity scores of subpopulation-specific genes in Treg subsets. Selected genes are indicated. **c** Violin plot showing the gene expression of *CD69*, *PRDM1*, *CXCR4*, *JUNB*, *DUSP2*, and *RELA* in MAIT cell cluster between HC and VKH group in scRNA-seq. For violin plots, two-sided Wilcoxon test was used to compared VKH groups versus HC groups. **d** Genome browser tracks showing single-cell chromatin accessibility in the *IFNG* loci in Th1 cells. **e** Genome browser tracks showing single-cell chromatin accessibility in the *IL10* loci in Treg cells. **f** Comparison of aggregate TF footprints for TBX21 and RUNX1 in Th17 cells from HC and VKH. **g** Comparison of aggregate TF footprints for EOMES and TBX21 in CD8 TEM cells from HC and VKH. All data are aligned and annotated to hg38 reference genome.

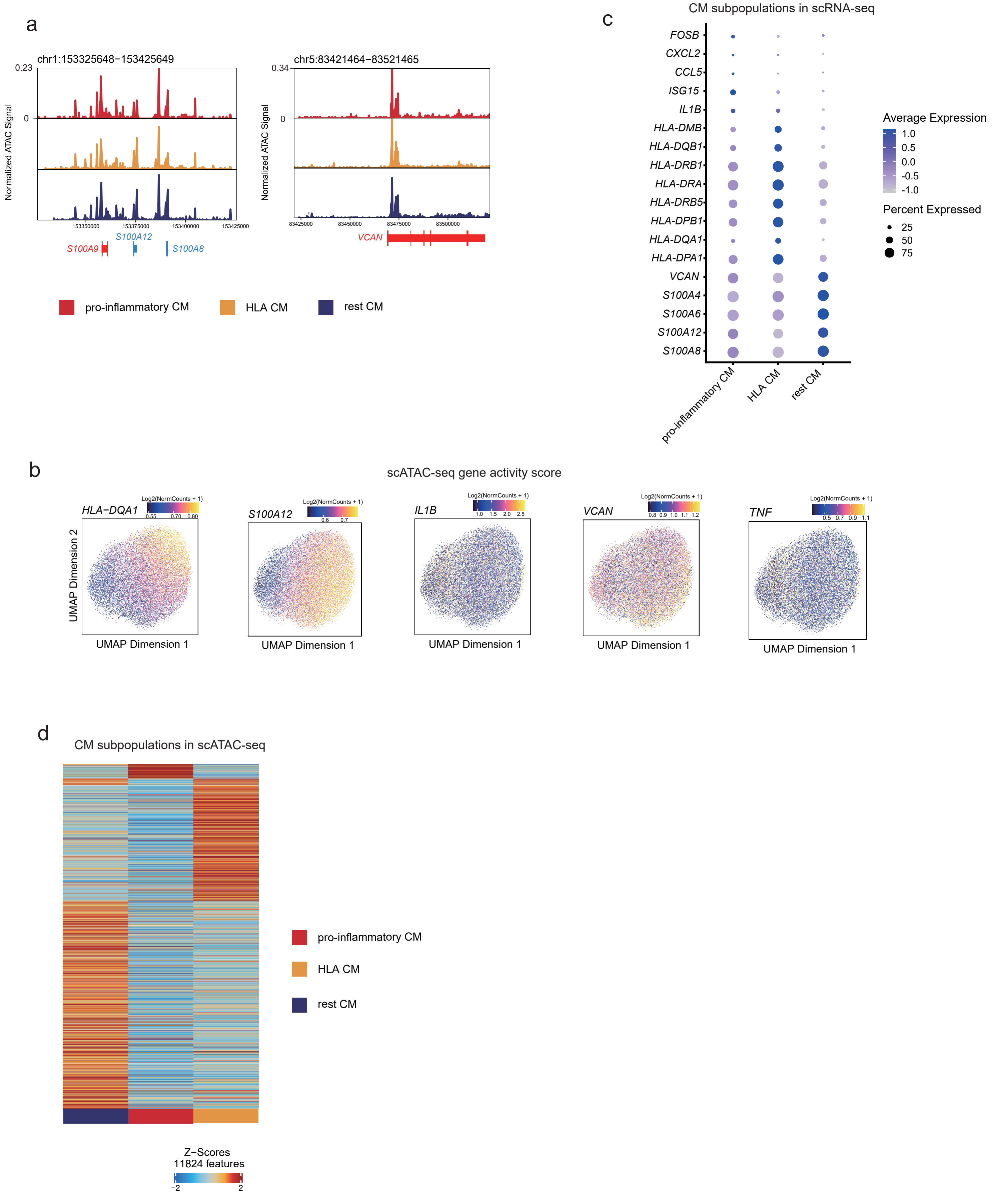
a
chr1:153325648−153425649
0.23
0
Normalized ATAC Signal
153350000
153375000
153400000
153425000
S100A12
S100A9
S100A8
chr5:83421464−83521465
0.34
0
Normalized ATAC Signal
83425000
83450000
83475000
83500000
VCAN
pro-inflammatory CM
HLA CM
rest CM
c
CM subpopulations in scRNA-seq
FOSB
CXCL2
CCL5
ISG15
IL1B
HLA-DMB
HLA-DQB1
HLA-DRB1
HLA-DRA
HLA-DRB5
HLA-DPB1
HLA-DQA1
HLA-DPA1
VCAN
S100A4
S100A6
S100A12
S100A8
pro-inflammatory CM
HLA CM
rest CM
Average Expression
1.0
0.5
0.0
-0.5
-1.0
Percent Expressed
25
50
75
b
scATAC-seq gene activity score
HLA−DQA1
S100A12
IL1B
VCAN
TNF
Log2(NormCounts + 1)
UMAP Dimension 2
UMAP Dimension 1
d
CM subpopulations in scATAC-seq
pro-inflammatory CM
HLA CM
rest CM
Z−Scores
11824 features
−2
2

Supplementary Figure 8. Anlysis of CM subgroups of scATAC-seq and scRNA-seq dataset.

a Genome browser tracks showing single-cell chromatin accessibility in the *S100A8* and *VCAN* locus. **b** UMAP projection of CM cell subgroup profiles colored by gene activity scores for the annotated lineage-defining genes in scATAC-seq dataset. The minimum and maximum gene activity scores are shown in each panel. **c** Dot plot of gene expression of the marker genes in CM subgroups. The dot size indicates the percentage of the cells in each cluster in which the gene of interest. The standardized gene expression level is indicated by color intensity. **d** Heatmap of Z-scores of 1,1824 *cis*-regulatory elements in scATAC-seq peripheral blood cell types derived from Fig. 5a. All data are aligned and annotated to hg38 reference genome.

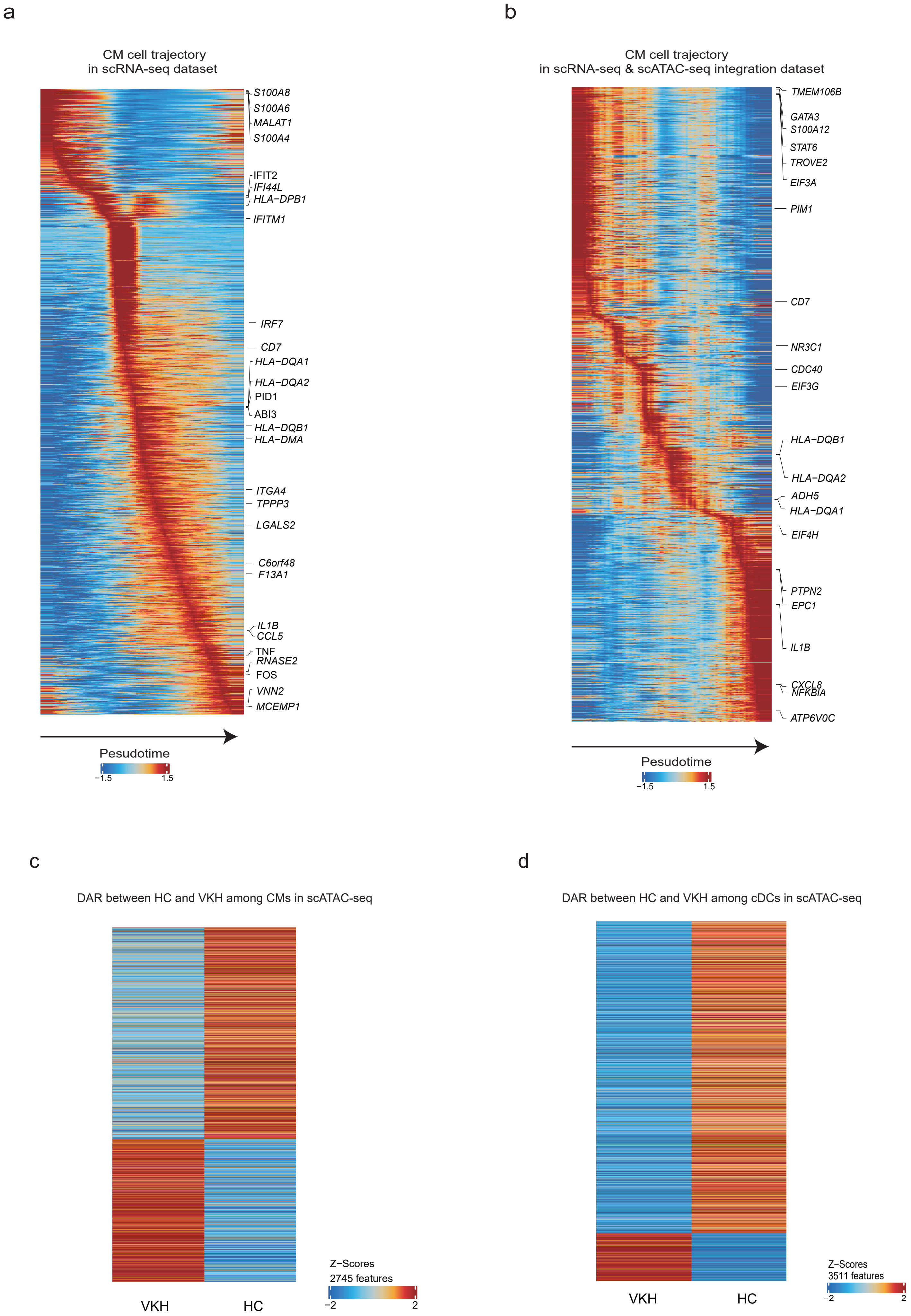
a
CM cell trajectory
in scRNA-seq dataset
S100A8
S100A6
MALAT1
S100A4
IFIT2
IFI44L
HLA−DPB1
IFITM1
IRF7
CD7
HLA−DQA1
HLA−DQA2
PID1
ABI3
HLA−DQB1
HLA−DMA
ITGA4
TPPP3
LGALS2
C6orf48
F13A1
IL1B
CCL5
TNF
RNASE2
FOS
VNN2
MCEMP1
Pesudotime
−1.5
1.5
b
CM cell trajectory
in scRNA-seq & scATAC-seq integration dataset
TMEM106B
GATA3
S100A12
STAT6
TROVE2
EIF3A
PIM1
CD7
NR3C1
CDC40
EIF3G
HLA−DQB1
HLA−DQA2
ADH5
HLA−DQA1
EIF4H
PTPN2
EPC1
IL1B
CXCL8
NFKBIA
ATP6V0C
Pesudotime
−1.5
1.5
c
DAR between HC and VKH among CMs in scATAC-seq
VKH
HC
Z−Scores
2745 features
−2
2
d
DAR between HC and VKH among cDCs in scATAC-seq
VKH
HC
Z−Scores
3511 features
−2
2

Supplementary Figure 9. Changes in myeloid cell subsets of scATAC-seq and scRNA-seq dataset among HC and VKH.

a Heatmaps of the ordered gene expression across pseudotime in the CM subgroups in scRNA-seq dataset (see Fig. 5d). **b** Heatmaps of the ordered gene integration expression across pseudotime in the CM subgroups in scRNA-seq and scATAC-seq integration dataset (see Fig. 5d). **c** Heatmap of Z-scores of DARs in CMs from HC and VKH. **d** Heatmap of Z-scores of DARs in cDCs from HC and VKH. All data are aligned and annotated to hg38 reference genome.

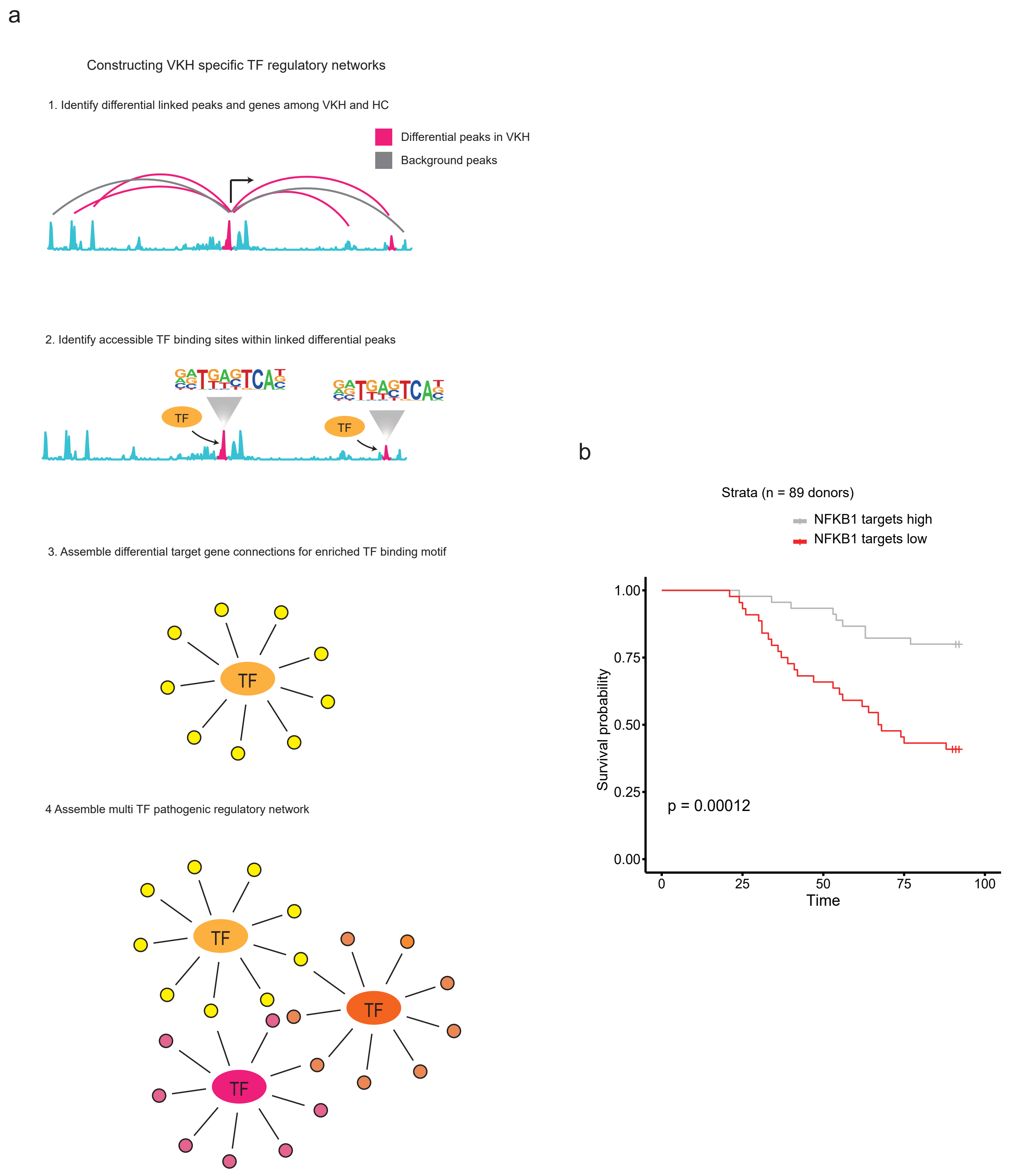

a
Constructing VKH specific TF regulatory networks
1. Identify differential linked peaks and genes among VKH and HC
Differential peaks in VKH
Background peaks
2. Identify accessible TF binding sites within linked differential peaks
TF
TF
3. Assemble differential target gene connections for enriched TF binding motif
TF
4 Assemble multi TF pathogenic regulatory network
TF
TF
TF
b
Strata (n = 89 donors)
NFKB1 targets high
NFKB1 targets low
Survival probability
1.00
0.75
0.50
0.25
0.00
p = 0.00012
0
25
50
75
100
Time

Supplementary Figure 10. Analysis of identification of TF target genes in cDCs in VKH patients.

a Schematic of construction of TF regulatory network. **b** Kaplan–Meier curve for patients with VKH (n = 89) stratified by putative NFKB1-target genes (n = 347); average z score log2(expression) (log-rank test *p* =0.0012). All data are aligned and annotated to hg38 reference genome.

Supplementary Table

Supplementary Table 1. Subjects' information in cohort 1.

Ident	Group	Gender	Age	Systemic Disease	scRNA-seq	scATAC-seq	Treatment
HC-1	HC	male	69	-	√	√	-
HC-2	HC	female	49	-	√	√	-
HC-3	HC	male	55	-	√	√	-
HC-4	HC	male	45	-	√	√	-
HC-5	HC	male	56	-	√	√	-
HC-6	HC	female	50	-	√	√	-
HC-7	HC	female	24	-	√	√	-
HC-8	HC	female	27	-	√	√	-
HC-9	HC	female	28	-	√	√	-
HC-10	HC	male	24	-	√	√	-
HC-11	HC	female	28	-	√	√	-
HC-12	HC	male	24	-	√	√	-
VKH-1	VKH	male	16	-	√	√	No Treatment
VKH-2	VKH	female	53	-	√	√	No Treatment
VKH-3	VKH	male	50	-	√	√	No Treatment
VKH-4	VKH	male	45	-	√	√	No Treatment
VKH-5	VKH	female	64	-	√	√	No Treatment
VKH-6	VKH	female	39	-	√	√	No Treatment
VKH-7	VKH	male	54	-	√	√	No Treatment
VKH-8	VKH	female	27	-	√	√	No Treatment
VKH-9	VKH	female	50	-	√	√	No Treatment
VKH-10	VKH	male	34	-	√	√	No Treatment
VKH-11	VKH	male	60	-	√	√	No Treatment
VKH-12	VKH	male	22	-	√	√	No Treatment

Supplementary Table 2. Gene sets used for cDCs Inflammation score calculation.

Gene
BST1
CD163
F13A1
S100A9
S100A8
VCAN
RNASE2
FCN1
CD14
TMEM176B
PLBD1
MGST1
RAB3D
CD36
