# Supplementary material for: Chromatin accessibility analysis reveals regulatory dynamics and therapeutic relevance of Vogt-Koyanagi-Harada disease
Source: Commun Biol. 2022 May 26;5:506. doi: 10.1038/s42003-022-03430-9 (PMC9135711; doi:10.1038/s42003-022-03430-9)
Supplement: Supplementary file 2 — Description of Additional Supplementary Files [file 42003_2022_3430_MOESM2_ESM.pdf]

Description of Additional Supplementary Files

File name: Supplementary Data 1.
Description: NF-kB family regulatory network in cDCs of VKH.

File name: Supplementary Data 2.
Description: Subjects' information in cohort 2.
