# Supplementary material for: Chromatin accessibility analysis reveals regulatory dynamics and therapeutic relevance of Vogt-Koyanagi-Harada disease
Source: Commun Biol. 2022 May 26;5:506. doi: 10.1038/s42003-022-03430-9 (PMC9135711; doi:10.1038/s42003-022-03430-9)
Supplement: Supplementary file 5 — Reporting summary [file 42003_2022_3430_MOESM5_ESM.pdf]

nature portfolio

Corresponding author(s): Yingfeng Zheng

Last updated by author(s): Apr 14, 2022

Reporting Summary

Nature Portfolio wishes to improve the reproducibility of the work that we publish. This form provides structure for consistency and transparency in reporting. For further information on Nature Portfolio policies, see our Editorial Policies and the Editorial Policy Checklist.

Statistics

For all statistical analyses, confirm that the following items are present in the figure legend, table legend, main text, or Methods section.

n/a	Confirmed	
☐	☒	The exact sample size (*n*) for each experimental group/condition, given as a discrete number and unit of measurement
☐	☒	A statement on whether measurements were taken from distinct samples or whether the same sample was measured repeatedly
☐	☒	The statistical test(s) used AND whether they are one- or two-sided<br>*Only common tests should be described solely by name; describe more complex techniques in the Methods section.*
☒	☐	A description of all covariates tested
☐	☒	A description of any assumptions or corrections, such as tests of normality and adjustment for multiple comparisons
☐	☒	A full description of the statistical parameters including central tendency (e.g. means) or other basic estimates (e.g. regression coefficient) AND variation (e.g. standard deviation) or associated estimates of uncertainty (e.g. confidence intervals)
☐	☒	For null hypothesis testing, the test statistic (e.g. *F*, *t*, *r*) with confidence intervals, effect sizes, degrees of freedom and *P* value noted<br>*Give P values as exact values whenever suitable.*
☒	☐	For Bayesian analysis, information on the choice of priors and Markov chain Monte Carlo settings
☒	☐	For hierarchical and complex designs, identification of the appropriate level for tests and full reporting of outcomes
☒	☐	Estimates of effect sizes (e.g. Cohen's *d*, Pearson's *r*), indicating how they were calculated

Our web collection on statistics for biologists contains articles on many of the points above.

Software and code

Policy information about availability of computer code

Data collection	Cell Ranger 3.1.0 - Barcode Identification, Alignment, Filter, Deduplication
Data analysis	We used publicly available softwares for all analysis and the data analysis was performed in R. These R packages listed in the methods with their appropriate citations and/or URLs. The code for TF network analysis in this study can be found on Github at https://github.com/GreenleafLab/MPAL-Single-Cell-2019. The code for plotting in this study can be found on https://gitlab.com/cvejic-group/integrative-scrnascatac-human-foetal

For manuscripts utilizing custom algorithms or software that are central to the research but not yet described in published literature, software must be made available to editors and reviewers. We strongly encourage code deposition in a community repository (e.g. GitHub). See the Nature Portfolio guidelines for submitting code & software for further information.

Data

Policy information about availability of data

All manuscripts must include a data availability statement. This statement should provide the following information, where applicable:

- Accession codes, unique identifiers, or web links for publicly available datasets
- A description of any restrictions on data availability
- For clinical datasets or third party data, please ensure that the statement adheres to our policy

All single-cell sequencing and bulk RNA sequencing data are available through the Beijing Institute of Genomics (BIG) under accession HRA001643.

The bulk ATAC-seq data were obtained from the Gene Expression Omnibus (GEO) under accession GSE137311 and GSE99702.

Field-specific reporting

Please select the one below that is the best fit for your research. If you are not sure, read the appropriate sections before making your selection.

☒ Life sciences ☐ Behavioural & social sciences ☐ Ecological, evolutionary & environmental sciences

For a reference copy of the document with all sections, see nature.com/documents/nr-reporting-summary-flat.pdf

Life sciences study design

All studies must disclose on these points even when the disclosure is negative.

Sample size	No sample size calculations were performed.<br><br>In cohort1, sample size for scATAC-seq and scRNA-seq (n=12 VKH; n=12 HC) was determined based on the knowledge on appropriate sample size to ensure adequate data for reliable assessment.<br><br>In cohort2, sample size for bulk RNA-seq (n=89 VKH) was determined based on the knowledge on appropriate sample size to ensure adequate data for reliable assessment.
Data exclusions	For scATAC-seq and scRNA-seq experiments, the details of our data quality control procedures are provided in the methods section of the manuscript.<br><br>For bulk RNA-seq, we performed quality control process included adapter trimming and low-quality read removal using Trim Galore (v0.6.4; https://github.com/FelixKrueger/TrimGalore) with parameters '—q 20 –phred 33 –stringency 3 –length 20 –e 0.1'.
Replication	All results presented in manuscript were reliably reproduced.
Randomization	No randomization was used in this study.
Blinding	No blinding was performed during data collection or analysis.

Reporting for specific materials, systems and methods

We require information from authors about some types of materials, experimental systems and methods used in many studies. Here, indicate whether each material, system or method listed is relevant to your study. If you are not sure if a list item applies to your research, read the appropriate section before selecting a response.

Materials & experimental systems

n/a	Involved in the study	
☒	☐	Antibodies
☒	☐	Eukaryotic cell lines
☒	☐	Palaeontology and archaeology
☒	☐	Animals and other organisms
☐	☒	Human research participants
☒	☐	Clinical data
☒	☐	Dual use research of concern

Methods

n/a	Involved in the study	
☒	☐	ChIP-seq
☒	☐	Flow cytometry
☒	☐	MRI-based neuroimaging

Human research participants

Policy information about studies involving human research participants

Population characteristics	In cohort1, 12 VKH patients (aged 16-65, 7 males and 5 females) and 12 healthy human subjects (aged 24-69, 6 males and 5 females) were recruited in the study. No significant differences in gender or age was detected between the two groups.<br><br>In cohort2, 89 VKH patients (aged 6-72, 38 males and 51 females) were recruited in the study.<br><br>For details on human samples used in this study, please see Table S1 and S4.
Recruitment	All healthy individuals and patients were recruited from Zhongshan Ophthalmic Center. Individuals with comorbid conditions including cancer, immunocompromising disorders, hypertension, diabetes, and steroid use were excluded.<br><br>VKH patients were recruited based on the diagnosis of VKH disease according to the revised diagnostic criteria established by the First International Workshop on VKH Disease. These patients showed initial active uveitis without any treatment before

blood drawing and were diagnosed based on history, systemic examination, and ocular features on indirect ophthalmoscopy, OCT, and FFA.

Ethics oversight	All study participants provided informed consent. The study was approved by The Ethics Committee of Zhongshan Ophthalmic Center (Guangzhou, China, 2019KYPJ114).

Note that full information on the approval of the study protocol must also be provided in the manuscript.
